# Can flipped classroom pedagogy offer promising perspectives for mathematics education on pandemic-related issues? A systematic literature review

**DOI:** 10.1007/s11858-022-01388-w

**Published:** 2022-06-28

**Authors:** Mustafa Cevikbas, Gabriele Kaiser

**Affiliations:** 1 University of Hamburg, Hamburg, Germany; 2 Nord University, Bodø, Norway

**Keywords:** Flipped classroom, COVID-19, Pandemic, Hybrid learning, Grand challenges and opportunities, Systematic review

## Abstract

Educators sometimes effect changes in education through the implementation of new ideas, and sometimes extraordinary circumstances force them to change their educational approaches, as during the COVID-19 crisis. Although we live in a digital age, the limited use of technology in education, particularly prior to the COVID-19 pandemic, and teachers’ insufficient experience with online or hybrid learning and teaching approaches resulted in several countries being unprepared for education during the pandemic. The flipped classroom (FC) is an innovative pedagogy with the potential to engage students in mathematics education using hybrid education combined with online and face-to-face learning, which is especially important during a pandemic. However, despite the high expectations surrounding this innovative approach, to date, no systematic literature review has discussed the opportunities and pitfalls of FCs in mathematics education regarding pandemic-related issues. In the present systematic review, we aim to bridge this gap and highlight the importance of flipping mathematics instruction during the pandemic and beyond. The results, which are based on textual analysis of 97 eligible articles, demonstrate that FC is a promising pedagogy that has numerous benefits for mathematics teaching and learning, although it is not a panacea for pandemic-related issues, as it also has several significant pitfalls. Overall, if the mechanism of mathematics education is to be crisis-ready, we should learn from experiences during the pandemic. In this regard, the current review contributes to research in mathematics education with the aim of gaining insight into successful implementations of FC pedagogy, not only during the pandemic but also beyond the crisis era of a pandemic.

**Supplementary Information:**

The online version contains supplementary material available at 10.1007/s11858-022-01388-w.

## Introduction

The exceptional situations caused by COVID-19 have made it necessary to change methods of teaching and learning all over the world. In particular, the pandemic has changed the agenda of mathematics education, turning students’ homes into their classrooms (Borba, 2021). This situation aligns with the flipped classroom (FC) pedagogy, an emerging pedagogical approach that enables effective use of technology and combines the advantages of face-to-face (f2f) and online instruction in order to engage students in active learning (Cevikbas & Kaiser, 2021). Recent studies in the field of mathematics education highlight that students and instructors have encountered various challenges related to online or distance learning during the pandemic, including a deficiency in communication and interaction between students and teachers or instructors (Borba, 2021), lack of learning motivation (Bakker et al., 2021), and growing anxiety (Bozkurt et al., 2020; Campillo-Ferrer & Miralles-Martinez, 2021). New structural considerations, teaching practices, and advocacy are needed in mathematics education to combat the problems that arose during the pandemic (NCSM & NCTM, 2020). Researchers have reported that blended/hybrid learning modes, particularly FC pedagogy, can provide opportunities during the pandemic for teachers and students (Bakker et al., 2021; Engelbrecht et al., 2020) to “redefine learning spaces, removing barriers between the home and school and making learning more accessible in a multiple of ways” (Attard & Holmes, 2020, p. 18). However, hardly any review has focused on the potential opportunities and pitfalls of applying FC pedagogy to improve mathematics education during and after the pandemic. Therefore, in this systematic literature review, we analyze this topic to uncover the potential of the FC approach in mathematics education during a crisis and beyond, based on the empirical results of prior studies.

## Background of the study

### Flipped classroom pedagogy

FC is an innovative pedagogy based on a hybrid mode of teaching that inverts traditional teaching methods, turning the spotlight from teachers to students, by providing lectures outside the classroom and performing active learning activities inside the classroom (Bergmann & Sams, 2012; Cevikbas & Kaiser, 2020). Although initial attempts were made to conceptualize FC in the early 2000s (Baker, 2000; Lage et al., 2000), there is still no consensus on the definition of FC. However, educators and researchers agree that FC is a student-centered pedagogy and that it has high potential to improve the quality of teaching and learning by freeing up class hours for social interaction, collaboration, inquiry, and deep learning (Cevikbas & Argün, 2017; Cevikbas & Kaiser, 2020, 2021).

Initial definitions refer to FC as homework at school and schoolwork at home (Baker, 2000; Bergmann & Sams, 2012; Lage et al., 2000), though the most recent definitions of FC go beyond this conceptualization (e.g., Bishop & Verleger, 2013; FLN, 2014). Bishop and Verleger’s (2013) definition identifies two components of FC, namely, interactive group learning activities inside the classroom and computer-based individual learning activities outside the classroom. This definition suggests that lecture and explanatory videos are a mandatory element of out-of-class activities, and that other types of materials (e.g., podcasts, slides, articles, lecture notes) are available along with the videos. Other descriptions for flipping the instruction include using quizzes, enhancing social interactions, performing question-and-answer sessions, and creating a technology-enhanced environment both in and out of the classroom (Abeysekera & Dawson, 2015; Cevikbas, 2018; Cevikbas & Kaiser, 2020, 2021; Talbert, 2015). In other variations of FC, students can watch videos or use other learning materials in the classroom, rather than out of the classroom (Howitt & Pegrum, 2015), or videos might be optional (Bergmann & Sams, 2012). These are especially common when not all students are able to access videos out of class because of technological and technical deficiencies.

The flipped learning network (FLN, 2014) developed a generic definition of FC, as follows:

Flipped learning is a pedagogical approach in which direct instruction moves from the group learning space to the individual learning space, and the resulting group space is transformed into a dynamic, interactive learning environment where the educator guides students as they apply concepts and engage creatively in the subject matter.

In order to meet the requirements of ‘flipped learning’ from the perspective of FLN, instructors have to encompass the four pillars of F-L-I-P (flexible environment, learning culture, intentional content, and professional educator) into their teaching (see FLN, 2014). Chen et al. (2014) added extra three letters, P-E-D (progressive activities, engaging experiences, and diversified platforms), to the F-L-I-P acronym for FC pedagogy in the higher education context. All of these definitions place greater emphasis on learners’ active roles and instructors’ design and guidance competencies. Another perspective on FC was provided by Staker and Horn (2012), who positioned FC under the rotational model of blended learning and described FC as comprising both f2f learning and online learning.

However, the COVID-19 pandemic crisis prompted researchers and educators to consider a change in this structure of FC, as it has not been possible to continue f2f instruction in most parts of the world. A new concept of FC combines synchronous and asynchronous online learning phases (Stöhr et al., 2020), expecting learners to complete pre-class tasks (e.g., watching videos) and be online for a class discussion. Jia et al. (2021) found that a fully online FC was as effective as a conventional FC for enhancing learners’ outcomes. The wide spectrum of pedagogical practices and the flexibility of FC pedagogy provide instructors with many possibilities for implementation, but little guidance on how to apply FC practices (Karabulut-Ilgu et al., 2018). Overall, from our point of view, instructors can take advantage of the rich theoretical perspectives and myriad implementation opportunities of FC pedagogy to engage students in active learning processes during a pandemic, which requires specific ways of teaching and learning, although a FC is not a silver bullet for pandemic-related issues in mathematics education.

### Previous survey studies on FCs

In the last decade, several review studies on FC pedagogy have been carried out in various research areas, such as engineering (e.g., Karabulut-Ilgu et al., 2018), nursing education (e.g., Tan et al., 2017), and educational sciences (e.g., Akcayir & Akcayir, 2018; Lo & Hew, 2017), and particularly in mathematics education (e.g., Fung et al., 2021; Lo et al., 2017; Yang et al., 2021). The majority of these reviews focus on the role of a FC in students’ academic performance (e.g., examination scores, achievement, learning progression) and their affective/emotional characteristics (motivation, satisfaction, self-efficacy/confidence, attitude, and perception).

Karabulut-Ilgu et al. (2018) analyzed 62 engineering education studies published up to 2015. The authors identified some benefits and challenges of FC pedagogy. The benefits included flexibility, interaction, and student engagement, and the challenges included workload for instructors and students, lack of live out-of-class sessions, technical problems, and decreased student interest.

Tan et al. (2017) conducted a meta-analysis of 29 studies published in 2015 and 2016 to examine the effectiveness of a FC in nursing education. They identified a significant post-intervention improvement in students’ achievement, satisfaction, attitude, critical thinking, self-learning abilities, and problem-solving skills. However, they did not report challenges associated with FCs.

Akcayir and Akcayir (2018) reviewed educational sciences articles published until 2016 in journals included in the Social Science Citation Index. Their analysis included 71 studies that identify the advantages and challenges of FC in educational sciences. Their results revealed that the most frequently cited advantage of a FC was improvement of student academic performance, followed by flexibility, interaction, satisfaction, engagement, motivation, critical thinking, pre-class preparation, autonomy, and collaboration. FC pedagogy was also associated with some difficulties, such as time consumption for students and teachers, limited student preparation, quality of videos, workload for students, problems with technology, lack of guidance out of class, adaptation problems, and student anxiety. Although this review revealed many advantages and challenges of FCs, the authors reported that their results were based on insufficient evidence to warrant generalization, and they called for future research on the advantages and disadvantages of FCs and traditional classrooms.

Lo and Hew (2017) conducted a systematic review of FCs in K-12 education and described a neutral or positive effect on student achievement and interaction, and mixed results concerning students’ perceptions and attitudes towards learning in FCs. They also reported some challenges associated with FCs, such as unfamiliarity with a FC, workload, problems with technology, lack of student preparation, and monitoring of students’ learning activities outside of the classroom.

In the field of mathematics education, although there is a relatively high number of studies on flipped mathematics classrooms, only a few systematic literature reviews have revealed state-of-the-art research on FCs from different perspectives. To date, no systematic review has addressed studies on flipped mathematics classes during the COVID-19 pandemic.

Lo et al. (2017) systematically reviewed 61 studies and conducted a meta-analysis of 21 studies published until 2016. From their results, they described several benefits of FCs, including more class time for active learning activities, accessibility of instructional videos, individualized learning, preparedness for in-class activities, peer-assisted learning, and immediate teacher feedback. The identified challenges included unfamiliarity with FC, lack of preparation for class hours, deficiency of live sessions out of class, increased workload, start-up effort, adaptation problems, and problems with technology. The limitations of this systematic review were as follows: (1) most of the reviewed studies largely concentrated on higher education; (2) the review focused solely on studies that contained lecture videos as part of the FC design; and (3) the duration of study interventions was not longer than one semester, which can be problematic due to the novelty effect.

Another systematic review study was conducted by Fung et al. (2021). The authors used ProQuest as database to identify 12 studies published from 2012 to 2017 on flipped mathematics classrooms. The authors found mixed results concerning the effect of FCs on students’ academic performance and perception. FC pedagogy produced relatively better academic results when the instructional design contained discussion, interaction, teacher feedback, and collaborative group work. Challenges included time-consuming activities, lack of teacher support out of class, and inconsistency between video content and in-class activities.

The study by Yang et al. (2021) focused on flipped mathematics classrooms and analyzed 82 articles published until 2018. Although the authors did not specifically examine the opportunities and pitfalls of FCs, their review yielded a few results on the benefits of FC pedagogy, in which FCs enhanced students’ learning performance, confidence, problem-solving skills, and attitudes towards learning.

Our systematic review study of the potential opportunities and pitfalls of FC in mathematics education is timely, as the popularity of FC pedagogy has increased in the field mathematics education (Yang et al., 2021). Additionally, although it remains a relatively new research field, the FC literature has witnessed rapid developments in recent years as described before. However, the potential of FCs in many content areas of mathematics has not yet been fully explored, and the studies described above do not reveal the actual potential of FC pedagogy on pandemic-related issues in mathematics. As witnessed not long ago, the COVID-19 pandemic has required a rapid transition in instructional methods from traditional modes to online and hybrid modes, including FC pedagogy. Therefore, there is a need to explore the potential of FCs not only for the pre-pandemic period, but also during the pandemic and beyond, as the FC pedagogy can be an opportunity to help teachers and students cope with pandemic-related demands for educational changes. At the same time, the pitfalls of FC should be known, in order to avoid unfavorable instructional experiences. Overall, to gain insight into successful implementations of FC pedagogy in mathematics education and to expose the pedagogy’s potential opportunities and pitfalls, it is necessary to conduct well-rounded systematic literature reviews that include literature published during the pandemic.

### Mathematics education during the pandemic

Although educators have attempted to develop and familiarize themselves with technological artifacts to integrate technology into mathematics education, actual implementations have lagged behind digital developments, especially before the COVID-19 pandemic (Cevikbas & Kaiser, 2020). As we highlighted earlier, the COVID-19 pandemic has changed the agenda of mathematics education and has resulted in the need to switch from f2f instruction to online instruction (Borba, 2021). This sudden change caught many mathematics educators off guard and brought with it many challenges.

Empirical evidence indicates that the pandemic has had negative impacts on students’ mental health, well-being, and academic growth, deepening pre-existing disparities in education (Goldberg, 2021). Furthermore, there are disturbing signs that some students may be lagging even further behind the achievements of the pre-pandemic era (Goldberg, 2021). Goldberg (2021) emphasized that many students in the U.S. context have been faced with challenges during the pandemic and have lost school-based professional support. Based on preliminary assessment data from the Curriculum Associates i-Ready platform in the U.S., Dorn et al. (2020) reported that crucial learning has been lost in mathematics. The students in their sample learned only 67% of the mathematics that they would normally have learned in previous years, equating to a loss of three to four months.

Although there is little empirical evidence of the actual impact of the COVID-19 pandemic on students’ learning in mathematics (Gore et al., 2021), similar challenges, including inequity and the digital divide, have been reported by researchers during the pandemic around the world (Bakker et al., 2021; Borba, 2021; Bozkurt et al., 2020; Engelbrecht et al., 2020; Gore et al., 2021). The most commonly cited challenges during the pandemic are related to accessing technological devices and the Internet, deficiency of communication and interaction between students and teachers, and assessing students’ learning progress. Sawchuk and Sparks (2020) highlighted that mathematics may be more sensitive to pandemic-related schooling disruptions than other subjects, for the following reasons. First, mathematics was formally learned in the classroom before the pandemic, and parents may not be able to provide professional support for their children to learn mathematics at home during the pandemic. Second, pandemic-related stress and trauma may intensify existing mathematics anxiety in some students. Third, it may be more challenging for instructors to provide effective mathematics instruction in remote learning environments.

Despite the challenges that have arisen during the pandemic, the COVID-19 crisis may create opportunities to foster digitalization and differentiated learning in mathematics education, as well as to enable creative practices and innovative pedagogies (Livy et al., 2021). Although COVID-19 restrictions have limited f2f education for a while, this situation has led to the implementation of myriad technology-supported learning/teaching approaches. Online, remote, and hybrid learning approaches, particularly FC pedagogy, which include digital tools, learning management systems (LMSs), lecture videos, massive open online courses, videoconferencing technologies, content-specific software such as Dynamic Geometry Software (DGS) and Computer Algebra Systems (CAS), and virtual manipulatives, may bolster effective learning in mathematics during the pandemic. Overall, as it is not known how long the COVID-19 pandemic will last or whether other pandemic crises will occur in the future, educators need to develop instructional principles to enhance effective mathematics learning in all possible conditions and prepare for crises in advance.

### Objectives of the study and research question

In this systematic review, we provide an overview of existing empirical studies, analyzing the potential of FC pedagogy in mathematics education based on empirically proven opportunities and pitfalls of FCs. Teaching activities during the pandemic were strongly related to flipping traditional teaching, as FCs contain both online and f2f instruction. In fact, FC pedagogy might have made teaching during the pandemic easier due to its flexible structure, which allows for switching between different instructional modes. The overarching goal of this study is to evaluate the potential of FCs to improve mathematics education during and after the COVID-19 pandemic and possible future pandemics. The following research question was addressed to explore evidence from the literature for the potential of FCs in mathematics education: What possibilities does FC pedagogy offer for mathematics education on pandemic-related issues?

The following section describes the methodology of the current systematic review study before we present our key results (opportunities and pitfalls of FC pedagogy in mathematics education) in detail. The paper concludes with a comprehensive discussion of the work based on the current systematic literature review, which may shed light on mathematics education during the pandemic and beyond.

## Methodology of the systematic review study

### Data sources and search strategies

We followed the Preferred Reporting Items for Systematic reviews and Meta-Analysis (PRISMA) guidelines (Moher et al., 2009) to structure our systematic review study. A literature search was performed on 16 June 2021. Systematic searches were conducted in the following electronic databases: (1) Web of Science, (2) Scopus, (3) ScienceDirect, and (4) Teacher Reference Center. We recruited these repositories as they have high-quality indexing standards and good international reputations, and they include studies in the field of mathematics education. To capture as many potentially relevant mathematics education research articles as possible, a diverse search request was developed to identify selected terms in articles’ titles, abstracts, and keywords (see Table 1).

**Table 1:** Search strings

Database	Search terms
Web of Science Core Collection	TOPIC: (flip* OR invert*) AND (class* OR learn* OR teach* OR instruction) AND (math*) Refined by: DOCUMENT TYPES: ( ARTICLE OR EARLY ACCESS) AND LANGUAGES: ( ENGLISH) AND RESEARCH AREAS: ( EDUCATION EDUCATIONAL RESEARCH OR SOCIAL SCIENCES OTHER TOPICS OR MATHEMATICS OR PSYCHOLOGY)
Scopus	TITLE-ABS-KEY ( ( flip* OR invert*) AND ( class* OR learn* OR teach* OR instruction) AND ( math*)) (LIMIT-TO (DOCTYPE, "ar") AND (LIMIT-TO (LANGUAGE, "English")) AND (LIMIT-TO (SUBJAREA, "MATH") OR LIMIT-TO (SUBJAREA, "SOCI") OR LIMIT-TO (SUBJAREA, "PSYC"))
ScienceDirect	(flipped OR inverted) AND (classroom OR learning OR teaching OR instruction) AND (math OR mathematics) (Refined by: review articles, research articles, discussion) AND (Refined by: social sciences, psychology, mathematics)
Teacher Reference Center	AB flip* learn* OR flip* class* OR flip* teach* OR flip* instruct* OR flip* math* OR invert* learn* OR invert* class* OR invert* teach* OR invert* instruct* AND math* Refined by source types: academic journals, reviews, case studies and Language: English

### Article selection criteria and procedure

The current study concentrates on English-language, peer-reviewed research articles published in the field of mathematics education whose results concern the opportunities or pitfalls of FCs in mathematics teaching and learning. Only empirical studies were included, as we were interested in the potential opportunities and pitfalls of FC pedagogy, which was—and is—of high relevance for teaching in pandemic times. We excluded editorials, book chapters, and conference proceedings, as these are not always peer-reviewed. Our search embraced studies conducted at all levels of mathematics education, with no restriction on publication year. The six inclusion criteria (IC) and six exclusion criteria (EC) we applied are presented in Table 2.

**Table 2:** Article selection criteria

Criteria	
Inclusion criteria (IC)	Exclusion criteria (EC)
IC1: Studies at all levels of mathematics education	EC1: Studies in a discipline other than mathematics education
IC2: Studies focus on FC implementations	EC2: FC is mentioned in the studies, but the focus is not on FC implementations
IC:3 Studies report results on opportunities or pitfalls of FC in mathematics education	EC3: Studies do not report any opportunities or pitfalls of FC in mathematics education
IC4: Peer-reviewed research articles	EC4: Editorials, books, book chapters, conference papers
IC5: The language of the studies is English	EC5: The language of the studies is not English
IC6: Studies indexed in Web of Science Core Collection, Scopus, ScienceDirect, or Teacher Reference Centre databases	EC6: Studies indexed in a database other than databases in the left column

**Fig. 1:**
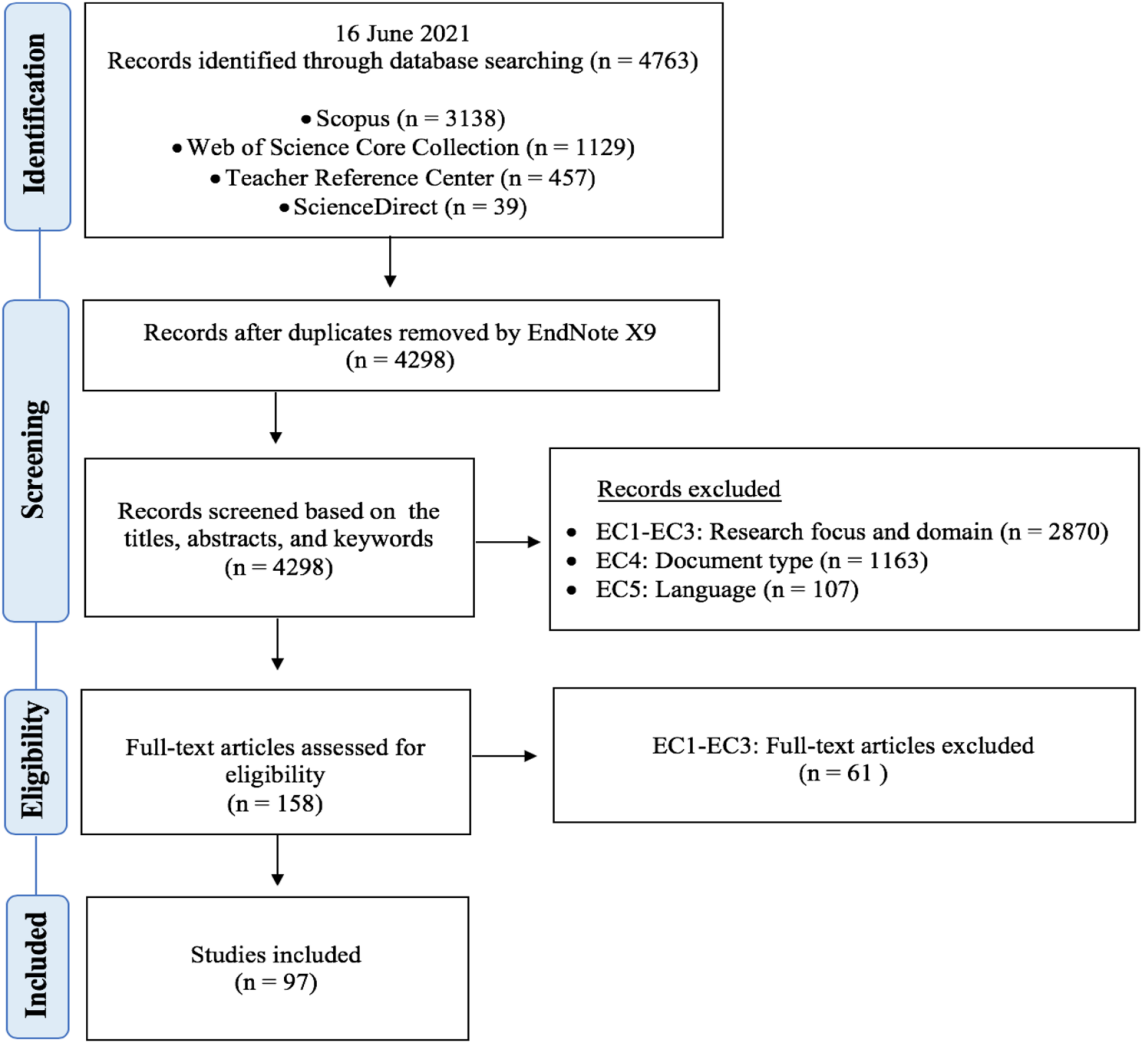
Flow chart of the article selection process

The article selection process comprised the four main steps of PRISMA: (1) identification, (2) screening, (3) eligibility, and (4) inclusion (Moher et al., 2009). First, the search strings from Table 1 were used to search four databases, identifying 4,763 papers. After removing 465 duplicated records with the help of EndNote X9 bibliographic software, we proceeded to the screening step. We carefully examined 4,298 papers’ titles, abstracts, and keywords based on our IC and EC, resulting in 158 potentially eligible articles. Then, we examined deeply the full-text versions of these articles based on the same IC and EC. Ultimately, we included 97 articles in the systematic review study (see the Appendix for the list of included articles and study characteristics). Figure 1 presents our article selection process, as strongly recommended by the PRISMA guidelines.

### Data analysis and reliability

For data analysis, we reviewed the full texts of all eligible articles and encoded them based on qualitative content analysis (Miles & Huberman, 1994), with the guidance of a newly developed coding scheme (see Table 5 in the Electronic Appendix), to access results that answered our research question. The analysis was structured around our research question, and coding concentrated on the following three main categories: (1) the main characteristics of the studies, (2) the opportunities of FCs, and (3) the pitfalls of FCs. Based on the results of these three categories, we discussed the potential of FC pedagogy during and after the pandemic for improving mathematics education. After conducting initial coding, 20% of the studies (n = 19) were randomly picked up and cross-checked for coherence by an external coder. Coding reliability was calculated based on Miles and Huberman’s (1994) reliability formula. The calculations produced a satisfactory reliability rate (0.91) according to Creswell (2013). Finally, the coders discussed any discrepancies and resolved them via consensus.

## Results of the study and discussion

In this systematic review we analyzed 97 research articles on the potential opportunities and pitfalls of FC pedagogy in mathematics education. The main results are provided below. (See the electronic appendix for the reviewed studies’ general characteristics.)

### Using instructional videos in FC studies

In our review, we examined the characteristics of instructional videos used in the studies individually. These videos are crucial elements in pre-class FC activities, and the content quality and main features of explanatory videos may affect the success of FC implementations during the COVID-19 pandemic. As the pandemic has caused global disruption to education systems (UNESCO, 2021) and restricted f2f learning, instructional videos are key in students’ mathematics learning. Studies have reported that students generally prefer short (less than 15 min) and teacher-generated videos because of their familiarity with the teacher’s instructional strategy (Akcayir & Akcayir, 2018; Cevikbas & Kaiser, 2020; Lo et al., 2017). Therefore, the videos’ sources and lengths are crucial in attracting students’ attention and may affect students’ video viewing rates during the pandemic.

Almost all flipped mathematics courses in the reviewed studies (n = 94) provided lecture videos before class as a means of sharing new content. In one study, students were given texts to read before class instead of watching pre-recorded videos. Two studies did not clarify whether lecture videos were used in flipped implementations. Our analysis revealed that several instructors (from 35 studies) preferred to create their own videos, while others (from 11 studies) used existing videos hosted on online platforms (e.g., Khan Academy, YouTube, HippoCampus, and MIT OpenCourseWare). Instructors in four studies used a mixture of their own and existing videos. Unfortunately, around half of the studies (n = 48) did not specify the sources of the instructional videos used in FC implementations. We also calculated the videos’ average lengths to determine the instructors’ preferences and that instructors used videos ranging from 1 to 40 min. In most studies, 1–10 min videos were used (n = 31), followed by 11–20 min videos (n = 20) and videos longer than 20 min (n = 3). Although several studies did not specify the videos’ precise lengths (n = 6), they stated that instructors used 3–20 min videos. A total of 36 studies did not provide detailed information about the video characteristics. Although instructional videos’ ideal lengths may vary depending on the target groups’ ages, most researchers recommend using videos shorter than 15 min, taking into account the individuals’ attention span (Bergmann & Sams, 2012; Cevikbas & Kaiser, 2020).

In summary, almost all flipped classroom instructors whose papers were included in our corpus preferred to use lecture videos when inverting their teaching both before and during the pandemic, although it is theoretically possible to provide content in different ways, such as through podcasts, infographics, reading texts, presentations, and lecture notes. This result confirmed the popularity of lecture videos—particularly instructor-created videos—in flipped mathematics classrooms, corroborating findings reported by Lo et al. (2017). According to Bishop and Verleger (2013), pre-class FC activities should include lecture videos, as many students find audiovisual resources more engaging than textual aids.

### Potential opportunities of flipped mathematics classrooms

Most studies (n = 94) provided empirical evidence regarding opportunities associated with flipped mathematics classrooms. We analyzed the opportunities offered by FCs (predominantly for the students, followed by instructors) reported in the reviewed studies in the context of five key categories (see Table 3) as follows: (1) academic development, (2) psychological and affective development, (3) social development, (4) meta-cognitive development, and (5) pedagogical development.

**Table 3:** Opportunities offered by flipped mathematics classrooms

Category	Sub-category	n^a^	Sample study^b^
Academic development	Achievement/performance/exam scores	40	Bhagat et al. (2016)
	Active learning, conceptual understanding	33	Love et al. (2015)
	Time-on-task	1	Cevikbas and Kaiser (2020)
Psychological and affective development	Perception, attitude	27	Turra et al. (2019)
	Enthusiasm, enjoying, entertaining	20	Johnston (2017)
	Learning motivation and interest	17	Chien and Hsieh (2018)
	Satisfaction and preferences	17	Nielsen et al. (2018)
	Self-efficacy/self-confidence	14	Yorganci (2020)
	Autonomy in learning	6	Belmonte et al. (2019)
	Feel comfortable	6	Patterson et al. (2018)
	Responsibility for learning, independent learning	5	Lopes and Soares (2018)
	Lower anxiety and stress	4	Weng (2015)
	Sense of competence	2	Lo and Hew (2020)
Social development	Collaboration, cooperation, teamwork	23	Dori et al. (2020)
	Interaction and dynamism	19	Cevikbas and Kaiser (2020)
	Discussion	8	Lo and Hew (2017)
	Communication	7	Karjanto and Simon (2019)
Meta cognitive development	Self-regulation	7	Lai and Hwang (2016)
	Diagnosing misconceptions and learning difficulties	6	Song (2020)
	Students’ mathematical/reflective thinking	5	Lee et al. (2017)
	Awareness	3	Guerrero et al. (2015)
	Visualization	2	Zengin (2017)
	Decision making	1	Belmonte et al. (2019)
	Metacognition	1	Naccarato and Karakok (2015)
	Accuracy	1	Mattis (2015)
	Reasoning	1	Fedistia et al. (2019)
Pedagogical development	Engagement, participation	28	Lo and Hew (2020)
	Freeing up class time, time management	17	Scott et al. (2016)
	Tailored instruction/learning, self-paced learning	16	Fedistia et al. (2019)
	Teacher feedback/scaffolding/support/guidance	12	Cevikbas and Kaiser (2020)
	Readiness, preparation for class hours	9	Lo and Hew (2020)
	Flexibility	3	Steen-Utheim and Foldnes (2018)
	Making more practices	2	Murphy et al. (2016)
	Equality	2	Grypp and Luebeck (2015)
	Transparency	1	Webel et al. (2018)
	Technology integration into mathematics	1	Cevikbas and Kaiser (2020)
	Paradigm shift	1	Cevikbas and Kaiser (2020)

^a^n represents the number of studies

^b^See the Appendix for a reference list of examples of studies

Our analysis indicates that the most commonly reported opportunities of FC pedagogy relate to its positive impact on students’ achievement (n = 40), learning progress (n = 33), engagement (n = 28), and collaborative/cooperative group work (n = 23). In general, the most widely cited category concerning FC opportunities is psychological and affective development (n = 118), followed by pedagogical development (n = 92), academic development (n = 74), social development (n = 57), and meta-cognitive development (n = 27). The relatively small number of studies reporting opportunities to foster students’ social development using FCs is unexpected due to the high importance of collaborative learning and social interaction in pedagogy during the pandemic.

As mentioned above, our review reveals that the most commonly reported opportunity for FC is the development of students’ achievement and learning performance in mathematics (undergraduates were dominant, followed by secondary school students). Almost half of the studies (n = 40) reported that FC had positive effects on students’ mathematics achievement/performance (i.e., an increase in students’ exam/test scores after FC interventions). More than one-third of the reviewed studies (n = 33) indicated that FC pedagogy improved students’ (conceptual) understanding and promoted active and continuous learning. These results demonstrate that FC significantly improved students’ academic development in mathematics, and that lecture videos and quizzes were the most effective elements of FC implementations.

Our findings regarding the positive effects of FC on active learning and students’ (conceptual) understanding confirm those of earlier survey studies. Most studies reviewed by Akcayir and Akcayir (2018) and Yang et al. (2021) reported relatively positive results with respect to student achievement, while Lo et al. (2017) reported neutral or positive results. Fung et al. (2021) emphasized that the evidence they obtained regarding students’ mathematics achievement was based solely on a comparison of FC pedagogy with traditional approaches, and that they excluded emerging pedagogies (e.g., virtual reality and augmented reality, simulations, adaptive learning, game-based learning). Comparison of our results with those of other, pre-pandemic surveys indicates that FC plays a primarily positive role in students’ mathematics achievement, and that this was the case both before and during the pandemic.

The most frequently reported opportunities with respect to students’ and teachers’ psychological and affective development were effects on the following: perceptions, attitudes, and feelings toward mathematics and FCs (n = 27); enjoyment, stimulation, and enthusiasm of FCs (n = 20); and learning motivation and interest (n = 17). The majority of studies reported that most students responded positively to the integration of FC components in mathematics learning and teaching. Consistent with these results, 17 studies reported that students were highly satisfied with their experiences of learning mathematics in FCs, and highlighted that students’ satisfaction and preference for learning mathematics in FC were due to the pedagogy’s flexibility, easy access to learning materials—particularly explanatory videos—and opportunities for active learning. Several teachers also reported high professional satisfaction with the FC approach in their teaching; the positive effects of FC on students’ achievement, self-confidence, and learning motivation made their teachers satisfied with FC implementations. Fewer studies reported reduced anxiety or the fostering of autonomy. Several studies reported positive effects on the psychological and affective development of students and teachers, which is promising for pandemic-era mathematics education, since students and teachers require motivation and supports to their well-being amid the challenges associated with the COVID-19 crisis (Goldberg, 2021).

Our analysis also indicated that FC provided opportunities for the social development of students and teachers, although these effects were reported in fewer studies than was the case for cognitive and affective effects. Almost one-third of the studies (n = 23) reported that FCs enhanced students’ abilities to collaborate and cooperate with their peers and actively participate in teamwork. Nineteen studies reported that FCs improved student–student and student–teacher interactions and increased dynamism in the learning/teaching process. Fewer studies reported that discussion and communication between students and their peers and teachers was promoted by watching lecture videos, taking notes, and engaging in group work both inside and outside the classroom. From a Vygotskian perspective, these results, which are consistent with those of earlier survey studies (Akcayir & Akcayir, 2018; Fung et al., 2021; Lo & Hew, 2017), can be interpreted as positive outcomes of FC, as knowledge and meaning can be constructed only through social interaction, which is significantly restricted in pandemic times (Cevikbas & Kaiser, 2020).

The influence of FCs on opportunities for meta-cognitive development was reported least frequently. Studies mentioned opportunities for fostering students’ self-regulation skills/strategies and teachers’ skills in diagnosing students’ misconceptions and learning difficulties. Teachers in FCs were able to gain information about students’ initial learning experiences before synchronous instruction through LMSs and activities, such as online discussions and question-and-answer sessions on content introduced through explanatory videos. Several studies reported that the rich design characteristics of FCs fostered students’ and teachers’ awareness of the different approaches to learning and teaching mathematics. These results indicate that FCs can foster both students’ and teachers’ meta-cognitive skills, which are crucial for the development of self-regulation strategies and diagnostic skills. The contribution of FCs to students’ meta-cognitive development may have supported them in overcoming the learning problems that they encountered during the COVID-19 pandemic, although the above-mentioned surveys yielded no findings regarding the role in this regard concerning students’ meta-cognitive skills.

Pedagogical development opportunities refer to developments facilitated by the theoretical design characteristics and innovative applications of FC pedagogy. Our analysis illustrated that the most cited pedagogical opportunity of a FC was its ability to foster students’ engagement (e.g., behavioral, cognitive, and emotional) and active participation in mathematics classrooms (n = 28). Several studies (n = 17) noted that FCs freed up class hours for active learning activities and made it easier for teachers to manage their time, providing them with opportunities to tailor their mathematics teaching and allowing students to learn at their own pace (n = 16). Several studies (n = 12) reported that teachers could provide more feedback and scaffolding in FCs than in traditional classrooms, and that students felt more supported and well-guided in FC implementations. Studies (n = 9) also found that students in FCs prepared in advance and felt equipped to delve more deeply into content in the classroom. The results confirm that the flexible design features of FCs are compatible with both online and f2f instruction and support the smooth shift from traditional approaches to online approaches during the pandemic.

### Potential pitfalls of flipped mathematics classrooms

In addition to the manifold benefits that FC offers for both students and instructors, several pitfalls emerge when inverting mathematics classrooms (see Table 4). Around half of the studies analyzed (n = 44) indicated that flipped mathematics classrooms were subject to several pitfalls. We classified these under the following four main categories: (1) pedagogical issues, (2) affective issues, (3) cognitive issues, and (4) technical issues.

**Table 4:** Pitfalls of flipped mathematics classrooms

Category	Sub-category	n^a^	Sample study^b^
Pedagogical issues	Workload, start-up effort, time-consuming tasks and activities	16	Muir and Geiger (2016)
	Difficulties in individual learning outside the classroom and personal responsibility	15	Adams and Dove (2018)
	Lack of preparation for class hours	12	Collins (2019)
	Adaptation problems and unfamiliarity with FC	6	Lo (2017)
	Engagement problems in group discussions	4	Johnston (2017)
	Difficulties in monitoring students’ learning progress	3	Cevikbas and Kaiser (2020)
	Low examination scores	1	Bego et al. (2020)
Technical issues	Problems with Internet connection and technology competency (use of technological tools, devices, software, etc.)	6	Zengin (2017)
	Unsuitable content for FC	4	Sen and Hava (2020)
Cognitive issues	Difficulties in remembering the content of the lecture videos	2	Lo and Hew (2017)
	Low task orientation	1	Strayer (2012)
Affective issues	Stress, anxiety, frustration	6	Strayer (2012)
	Lack of motivation	4	Tse et al. (2019)
	Dissatisfaction	3	Bagley (2020)
	Being bored	3	Guerrero et al. (2015)
	Negative attitude	2	Cilli-Turner (2015)

^a^n represents the number of studies

^b^See the Appendix for a reference list of examples of studies

The most commonly reported pitfalls of FCs were pedagogical issues, including start-up effort, workload (mainly for instructors and partially for students), and time-consuming activities (n = 16), as well as difficulties in pre-class individual learning (n = 15). In FCs, instructors must plan lessons and create new content or adapt pre-existing content to enable students to learn relevant topics and prepare for class hours. Almost all the reviewed studies’ FC designs contained lecture videos, which required time to create or identify on existing platforms. In addition, students in FCs invested significant effort in understanding new topics individually before class hours and completed extra pre-class tasks, such as watching lecture videos (in most cases) or reading text-based documents and taking notes. Students in several studies reported that the lack of live sessions outside the classroom and deficiency of simultaneous interaction while watching videos were pitfalls of FCs, as the differentiation in learning led to increased personal responsibility for students. Another pitfall was students’ poor preparation for lessons (n = 12), which negatively affected the quality of the in-class experience. For instance, when students did not watch the explanatory videos in advance, they could not actively engage in discussions and group work in the classroom (n = 4). Moreover, some students were unfamiliar with FC pedagogy or/and struggled to adapt (n = 6). Radical changes in learning routines may be challenging for students, and consequently, they may prefer traditional classroom formats. FC pedagogy may become even more difficult during periods of crisis, as considerable effort and time are required to overcome such structural challenges.

Earlier studies from educational science and other disciplines supported our findings regarding the pedagogical pitfalls for students and/or teachers in mathematics education. These studies reported start-up efforts for teachers, high workloads for students and teachers, time-consuming activities for students and teachers, limited student preparation, students’ lack of familiarity with FCs, students’ adaptation problems, and lack of live sessions and guidance outside the classroom (Akcayir & Akcayir, 2018; Fung et al., 2021; Karabulut-Ilgu et al., 2018; Lo & Hew, 2017; Lo et al., 2017). Attard and Holmes (2020) reported similar potential pitfalls for blended learning approaches in general.

In our review, several studies (n = 6) reported pitfalls associated with technical problems, such as Internet connection problems (for students) and the need to use technological devices and software to produce instructional materials, such as explanatory videos (for instructors). These results align with those of other survey studies (Akcayir & Akcayir, 2018; Karabulut-Ilgu et al., 2018; Lo & Hew, 2017). In addition, not all mathematics topics were appropriate for students to learn independently online, and some instructors struggled to teach complicated content through videos (n = 4). Although these technical shortcomings have been reported in a limited number of studies, these problems may undermine the implementation of FCs, particularly during crisis periods, as there is no possibility of compensating for online teaching during such times.

Our analysis revealed that cognitive issues were not associated with many pitfalls for FC pedagogy. Two main pitfalls emerged: (1) students could not remember what they learned via lecture videos or other materials when they returned to the classroom, as these activities might have been completed several days before (n = 2), and (2) lower task orientation (n = 1). Although these results were limited, they should be addressed through the introduction of short warm-up activities at the beginning of live instruction to help students concentrate on the topic and learning activities during the pandemic.

In our review we found that several studies reported affective problems for students and instructors in FCs. Most of these problems were associated with the following elements: anxiety, stress, and frustration about the new learning environment; tasks; activities; exams/quizzes; lecture videos; and collaborative group work (n = 6). Other reported pitfalls were students’ and instructors’ lack of pre-class motivation (n = 4), followed by students’ dissatisfaction with learning in FCs (n = 3), activities that were boring from both the teachers’ and students’ perspectives (n = 3), and negative attitudes toward time-consuming activities (n = 2). Other survey studies found that students may experience anxiety in FCs owing to the new learning environment, responsibilities, and tasks (Akcayir & Akcayir, 2018), which may threaten students’ well-being amid the pandemic crisis. To sum up, fewer pitfalls than opportunities have been reported in relation to FC, but teachers must nonetheless be aware of these, not only, but especially, during periods of crisis, such as the COVID-19 pandemic.

## Looking ahead—flipping mathematics instruction during a pandemic and beyond

This systematic review study focused on FC’s empirically proven opportunities and pitfalls to uncover the potential and use of FCs in relation to mathematics education, particularly during the global pandemic and beyond. The distribution of the opportunities and pitfalls of FC identified in our review indicated that the opportunities predominate. This is a promising finding, as it implies that mathematics education can benefit from FC both during and after the COVID-19 pandemic and during potential future crisis events. Instructional activities during the pandemic aligned closely with FC pedagogy, and full adoption of the FC approach may make pandemic-era teaching easier by virtue of its flexible structure and potential to switch between different instructional modes. The flexible structure can be adapted to pandemic requirements owing to the synergy between the main characteristics of FC principles and the pandemic-related restraints of online learning (Foster et al., 2022; Swart et al., 2022): “At this point in the pandemic, it appears that attempts have favored replicating as closely as possible a ‘normal’ classroom, and the more traditional the classroom, the more difficult this is to reproduce meaningfully in an online context” (Foster et al., 2022, p. 12). Swart et al.’s (2022) findings suggest that students with experience of FCs are well prepared for crises, as they are familiar with online learning and teachers already have adequate online instructional materials. FC pedagogy appears to have worked well during the pandemic, particularly where it had already been introduced prior to the crisis (Divjak et al., 2022).

Apparently, no instructional system can be developed to be pandemic-proof; however, amid the uncertainty caused by the COVID-19 crisis, it is crucial to investigate what may be required to build greater robustness and crisis-preparedness into mathematics education in schools and universities (Foster et al., 2022). The instructional approach must be attractive to students, acceptable to teachers, and robust in use (Foster et al., 2022). In meeting these criteria, FC pedagogy may play an important role in improving mathematics education during crises. In this section, we focus on the main challenges that students and teachers encountered during the pandemic and which opportunities connected with FCs are reported as addressing pandemic-related problems; we further discuss the role played by FCs in mathematics education during the pandemic, considering both its opportunities and pitfalls.

Many students and teachers faced critical problems in mathematics education with the onset of the pandemic. For example, Hodgen et al. (2020) reported that the COVID-19 pandemic limited most students’ opportunities to learn mathematics and caused significant learning loss. They also stressed that the pandemic has restricted (a) professional support via scaffolding for students, (b) engagement in mathematical talks, (c) participation in meta-cognitive activities, (d) opportunities for formative feedback, and (e) interactions with teachers and peers in the learning process. In addition, Cao et al. (2021) identified the following challenges for mathematics teachers during the pandemic: (a) the use of technology, (b) classroom management and difficulties in monitoring student engagement, (b) adapting to the shift in teacher-student interactions within online teaching modes, (c) preparing adequate materials for online instruction, and (d) being flexible with lesson design. This crisis has also widened the digital divide through differences in technological provision and home circumstances (Foster et al., 2022) and deepened the socioeconomic achievement gap, namely, inequality among students (Hodgen et al., 2020).

Our systematic survey’s positive findings imply that FC may mitigate the negative and devastating impact of these pandemic-related issues on students and teachers. As expected, many researchers have focused on FC pedagogy’s impact on students’ achievement and learning performance, and several studies completed both prior to and during the pandemic have reported positive results. This result is particularly salient during the pandemic, as the cumulative and hierarchical nature of mathematics makes students particularly vulnerable to the disruption caused by COVID-19 (Foster et al., 2022). In particular, the use of explanatory videos, communication through LMSs, the creation of dynamism and interaction in the learning environment, and collaborative group work are important elements of FC implementation that improve the academic development of mathematics students and may mitigate the pandemic’s destructive impact on student achievement. However, our findings indicate that the positive influence of FC pedagogy on students’ academic development largely depends on their self-discipline and learning responsibilities. If learners are poorly prepared and have not fully completed their asynchronous learning activities (e.g., watching videos, using reading materials, taking notes), they may struggle with synchronous mathematics activities, which will in turn negatively impact their mathematics achievement amid the COVID-19 crisis (Cao et al., 2021).

As noted, the COVID-19 pandemic has threatened socialization and well-being in education, hindered interaction, and limited modes of communication (Cevikbas & Kaiser, 2020; Goldberg, 2021). FC pedagogy’s opportunities for the social and affective development of students and teachers have been reported in several studies conducted both before and during the pandemic. Our results indicate that FC pedagogy has the potential to improve individuals’ well-being in mathematics education and to break down the walls of classrooms by promoting technology-enabled socialization. Through technology-supported approaches, educators can reopen the communication paths that were blocked by the necessary disruption to in-person schooling, which is crucial for students, during the COVID-19 pandemic (Borba, 2021; Engelbrecht et al., 2020). The most recent studies conducted during the pandemic identified problems in students’ engagement in learning associated with the fact that students were more socially isolated, received less social and pedagogical support, and were at greater risk of developing mental health problems, all of which have the potential to negatively affect their engagement in learning (Koob et al., 2021). Amid such pandemic-related problems, the culture of students’ (and parents’) engagement offered by FC pedagogy may provide crucial support. Consistent with these results, Swart et al. (2022) reported that students with FC experience were more resilient, more engaged, and more satisfied than students without FC experience when required to transition from f2f to online learning. Furthermore, COVID-19 restricted participants’ meta-cognitive activities owing to missing regulation possibilities (Hodgen et al., 2020). In an FC learning environment, students may develop their meta-cognitive strategies by engaging in technologically rich activities during pandemic times.

Although empirical studies have confirmed the manifold opportunities of FCs in mathematics education during the pandemic, the pedagogical pitfalls of this pedagogy remain to be fully considered. As the empirical studies from our review highlight, FC pedagogy is challenging, particularly during the initial stages of flipped instruction, as instructors must frequently create new content (e.g., videos) and learners are tasked with new pre-class learning responsibilities (Bergmann & Sams, 2012; Cevikbas, 2018), which may not be manageable without professional support, which is difficult to get within the COVID-19 pandemic. In the long run, in light of the potential longevity and uncertainty of pandemics, instructors should regularly use videos and other instructional materials prepared in advance, making necessary improvements based on developments in the subject-specific fields (Cevikbas, 2018; Cevikbas & Argün, 2017). To prepare for such hybrid teaching scenarios, instructors must collaborate with their colleagues and experts on content creation to reduce their workloads.

The analyzed studies also reported several structural and technical pitfalls of FC, such as Internet connection problems, deficiencies in the use of digital tools, mobile devices, and software, and the need to create new content. These deficiencies have been among the most significant pitfalls of FC during the COVID-19 crisis, despite considerable efforts to facilitate use by students, parents, instructors, and institutions, including the provision of tips, recommendations, useful resources, and guidelines (Bozkurt et al., 2020; UNESCO, 2021). In addition, the affective pitfalls of FC are particularly important, as the COVID-19 pandemic may lead to increased stress and anxiety during the learning process (Bozkurt et al., 2020; Hofer et al., 2021). Although it has been reported only in a small number of studies, some students’ and teachers’ emotional development may be negatively affected in FCs, among other causes by their lack of familiarity with FCs, which may pose additional challenges during periods of crisis, as Swart et al. (2022) confirmed.

To summarize, FC pedagogy, as a hybrid learning mode, offers several possibilities for mathematics teaching and learning during pandemics. Such possibilities concern not only affective and pedagogical aspects but also content-related issues, such as the possibility of introducing new mathematical content via explanatory videos. We hope that our systematic analysis of the literature on FC and its potential for mathematics education will encourage educators and researchers to consider developing innovative FC designs and creative applications so that no student’s learning is hindered during the pandemic. In a post-pandemic world, educators will be well positioned to take advantage of the key soft skills gained during the COVID-19 pandemic, increasing the likelihood that FC pedagogy or similar emerging pedagogies will become more prevalent in the future (Attard & Holmes, 2020).

This systematic review study––building on previous empirical studies––is one of several other important studies in the growing body of FC research in mathematics education that specifically calls on learners, teachers, researchers, and policymakers to equip themselves for global pandemic crises and their aftermaths. If our mathematics education systems are to be crisis-ready, we must learn from events during the COVID-19 crisis and ensure that we are better prepared for the future (Foster et al., 2022). Although FC pedagogy is not a panacea for pandemic-related issues, this pedagogy may offer tremendous opportunities for mathematics education, particularly during crisis periods.

## Limitations

Although we applied PRISMA guidelines to improve the transparency, accuracy, and quality of the study, and our search strategy was extensive, the study had several limitations with respect to its selection criteria. We concentrated on peer-reviewed journal articles that were published in English and indexed in high-ranking databases. According to our criteria, potentially eligible studies had to focus on and provide empirical results regarding the opportunities and pitfalls of FCs. These selection criteria may have resulted in the exclusion of FC pedagogy studies that focused indirectly on these aspects. Another limitation may relate to the “jingle-jangle fallacy” (Gonzalez et al., 2021) of automated study selection. In other words, the titles, abstracts, or keywords of certain studies may not have included words that matched the search strings we used to carry out this systematic review, even if they focused on the opportunities and pitfalls of FC pedagogy. Although we obtained a relatively large sample, future studies may consider broadening their focus and using different databases. Moreover, as many of the studies did not clarify when their data were collected, we could not clearly distinguish between the results of studies conducted before or during the pandemic. Another possible bias may be related to the reliability of the reviewed studies’ results, as most used short-term FC interventions (e.g., one academic semester or less) and were based on a small sample size (e.g., fewer than 100 participants). This means that some results regarding the opportunities and pitfalls of FCs may have differed positively or negatively across short- and long-term interventions. Further studies are required to overcome these limitations.

## Supplementary Information

Below is the link to the electronic supplementary material.

Supplementary file1 (PDF 6157 KB)

## References

[CR1] Abeysekera L, Dawson P (2015). Motivation and cognitive load in the flipped classroom: Definition, rationale and a call for research. Higher Education Research and Development.

[CR47] Adams Caleb (2018). Calculus students flipped out: The impact of flipped learning on calculus students' achievement and perceptions of learning. Primus.

[CR2] Akcayir G, Akcayir M (2018). The flipped classroom: A review of its advantages and challenges. Computers & Education.

[CR3] Attard C, Holmes K (2020). An exploration of teacher and student perceptions of blended learning in four secondary mathematics classrooms. Mathematics Education Research Journal.

[CR48] Bagley Spencer (2020). The flipped classroom, lethal mutations, and the didactical contract: A cautionary tale.. Primus.

[CR4] Baker, J. W. (2000, April). The “classroom flip”: Using web course management tools to become a guide by the side [Paper presentation]. In *11th international conference on college teaching and learning*, Jacksonville.

[CR5] Bakker A, Cai J, Zenger L (2021). Future themes of mathematics education research: An international survey before and during the pandemic. Educational Studies in Mathematics.

[CR50] López Belmonte Jesús (2019). Formative transcendence of flipped learning in mathematics students of secondary education. Mathematics.

[CR49] Bego Campbell Rightmyer (2020). Improving performance in a large flipped barrier mathematics course: A longitudinal case study. International Journal of Mathematical Education in Science and Technology,.

[CR6] Bergmann, J., & Sams, A. (2012). *Flip your classroom: Reach every student in every class every day*. ISTE.

[CR51] Bhagat KK (2016). The impact of the flipped classroom on mathematics concept learning in high school.. Journal of Educational Technology & Society.

[CR7] Bishop JL, Verleger MA (2013). The flipped classroom: A survey of the research. ASEE National Conference Proceedings.

[CR8] Borba MC (2021). The future of mathematics education since COVID-19: Humans-with-media or humans-with-non-living-things. Educational Studies in Mathematics.

[CR9] Bozkurt A, Jung I, Xiao J, Vladimirschi V, Schuwer R, Egorov G, Paskevicius M (2020). A global outlook to the interruption of education due to COVID-19 pandemic: Navigating in a time of uncertainty and crisis. Asian Journal of Distance Education.

[CR10] Campillo-Ferrer JM, Miralles-Martinez P (2021). Effectiveness of the flipped classroom model on students’ self-reported motivation and learning during the COVID-19 pandemic. Humanities and Social Sciences Communications.

[CR11] Cao Y, Zhang S, Chan MCE, Kang Y (2021). Post-pandemic reflections: Lessons from Chinese mathematics teachers about online mathematics instruction. Asia Pacific Education Review.

[CR12] Cevikbas, M. (2018). *Ters-yüz sınıf modeli uygulamalarına dayalı bir matematik sınıfındaki öğrenci katılım sürecinin incelenmesi* [Investigation of student participation process in a mathematics classroom based on flipped classroom model applications] [Unpublished doctoral dissertation]. Gazi University.

[CR13] Cevikbas M, Argün Z (2017). An innovative learning model in digital age: Flipped classroom. Journal of Education and Training Studies.

[CR14] Cevikbas M, Kaiser G (2020). Flipped classroom as a reform-oriented approach to teaching mathematics. ZDM Mathematics Education.

[CR15] Cevikbas M, Kaiser G (2021). Student engagement in a flipped secondary mathematics classroom. International Journal of Science and Mathematics Education.

[CR52] Chien Chih-Feng, Hsieh Lin-Han Chiang (2018). Exploring University Students' Achievement, Motivation, and Receptivity of Flipped Learning in an Engineering Mathematics Course. International Journal of Online Pedagogy and Course Design.

[CR53] Cilli-Turner Emily (2015). Measuring Learning Outcomes and Attitudes in a Flipped Introductory Statistics Course. PRIMUS.

[CR16] Chen Y, Wang Y, Chen NS (2014). Is FLIP enough? Or should we use the FLIPPED model instead?. Computers & Education.

[CR54] Collins Benjamin V. C. (2019). Flipping the precalculus classroom. International Journal of Mathematical Education in Science and Technology,.

[CR17] Creswell JW (2013). Qualitative inquiry and research design: Choosing among five approaches.

[CR18] Divjak B, Rienties B, Iniesto F, Vondra P, Žižak M (2022). Flipped classrooms in higher education during the COVID-19 pandemic: Findings and future research recommendations. International Journal of Educational Technology in Higher Education.

[CR55] Dori Yehudit Judy, Kohen Zehavit (2020). Mathematics for computer science: A flipped classroom with an optional project. EURASIA Journal of Mathematics, Science and Technology Education.

[CR19] Dorn E, Hancock B, Sarakatsannis J, Viruleg E (2020). COVID-19 and learning loss—Disparities grow and students need help.

[CR20] Engelbrecht J, Borba MC, Llinares S, Kaiser G (2020). Will 2020 be remembered as the year in which education was changed?. ZDM Mathematics Education.

[CR56] Fedistia R (2019). Advantages and challenges of the flipped classroom application based learning in enhancing 10th grade senior high school students’ reasoning ability. International Journal of Scientific & Technology Research,.

[CR21] FLN. (2014). *Definition of flipped learning*. Flipped Learning Network. https://flippedlearning.org/definition-of-flipped-learning/

[CR22] Foster C, Burkhardt H, Schoenfeld A (2022). Crisis-ready educational design: The case of mathematics. The Curriculum Journal.

[CR23] Fung CH, Besser M, Poon KK (2021). Systematic literature review of flipped classroom in mathematics. Eurasia Journal of Mathematics, Science and Technology Education.

[CR24] Goldberg, S. B. (2021). *Education in a pandemic: The disparate impacts of COVID-19 on America’s students*. U.S. Department of Education, Office for Civil Rights. https://www.gcedclearinghouse.org/resources/education-pandemic-disparate-impacts-covid-19-america%E2%80%99s-students

[CR25] Gonzalez O, MacKinnon DP, Muniz FB (2021). Extrinsic convergent validity evidence to prevent jingle and jangle fallacies. Multivariate Behavioral Research.

[CR57] Grypp Lynette, Luebeck Jennifer (2015). Rotating Solids and Flipping Instruction. The Mathematics Teacher.

[CR58] Guerrero Shannon, Beal Melissa, Lamb Chris, Sonderegger Derek, Baumgartel Drew (2015). Flipping Undergraduate Finite Mathematics: Findings and Implications. PRIMUS.

[CR26] Gore J, Fray L, Miller A, Harris J, Taggart W (2021). The impact of COVID-19 on student learning in New South Wales primary schools: An empirical study. Australian Educational Research.

[CR27] Hodgen, J., Taylor, B., Jacques, L., Tereshchenko, A., Kwok, R., & Cockerill, M. (2020). *Remote mathematics teaching during COVID-19: Intentions, practices and equity.* University College London Institute of Education.

[CR28] Hofer SI, Nistor N, Scheibenzuber C (2021). Online teaching and learning in higher education: Lessons learned in crisis situations. Computers in Human Behavior.

[CR29] Howitt C, Pegrum M (2015). Implementing a flipped classroom approach in postgraduate education: An unexpected journey into pedagogical redesign. Australasian Journal of Educational Technology.

[CR30] Jia C, Hew KF, Bai S, Huang W (2021). Adaptation of a conventional flipped course to an online flipped format during the Covid-19 pandemic: Student learning performance and engagement. Journal of Research on Technology in Education.

[CR59] Johnston Barbara M. (2017). Implementing a flipped classroom approach in a university numerical methods mathematics course. International Journal of Mathematical Education in Science and Technology.

[CR31] Karabulut-Ilgu A, Jaramillo Cherrez N, Jahren CT (2018). A systematic review of research on the flipped learning method in engineering education. British Journal of Educational Technology.

[CR60] Karjanto N., Simon L. (2019). English-medium instruction Calculus in Confucian-Heritage Culture: Flipping the class or overriding the culture?. Studies in Educational Evaluation.

[CR32] Koob C, Schröpfer K, Coenen M, Kus S, Schmidt N (2021). Factors influencing study engagement during the COVID-19 pandemic: A cross-sectional study among health and social professions students. PLoS ONE.

[CR61] Lai Chiu-Lin, Hwang Gwo-Jen (2016). A self-regulated flipped classroom approach to improving students’ learning performance in a mathematics course. Computers & Education.

[CR33] Lage MJ, Platt GJ, Treglia M (2000). Inverting the classroom: A gateway to creating an inclusive learning environment. The Journal of Economic Education.

[CR62] Lee Jihyun, Lim Cheolil, Kim Hyeonsu (2017). Development of an instructional design model for flipped learning in higher education. Educational Technology Research and Development.

[CR34] Livy S, Muir T, Murphy C, Trimble A (2021). Creative approaches to teaching mathematics education with online tools during COVID-19. International Journal of Mathematical Education in Science and Technology.

[CR63] Lo Chung Kwan (2017). Examining the Flipped Classroom through Action Research. The Mathematics Teacher.

[CR35] Lo CK, Hew KF (2017). A critical review of flipped classroom challenges in K-12 education: Possible solutions and recommendations for future research. Research and Practice in Technology Enhanced Learning.

[CR200] Lo CK, Hew KF (2020). A comparison of flipped learning with gamification, traditional learning, and online independent study: The effects on students’ mathematics achievement and cognitive engagement. Interactive Learning Environments.

[CR36] Lo CK, Hew KF, Chen G (2017). Toward a set of design principles for mathematics flipped classrooms: A synthesis of research in mathematics education. Educational Research Review.

[CR64] Lopes Ana Paula (2018). Perception and performance in a flipped Financial Mathematics classroom.. The International Journal of Management Education.

[CR201] Love B, Hodge A, Corritore C, Ernst DC (2015). Inquiry-based learning and the flipped classroom model. Primus.

[CR65] Mattis Kristina V. (2015). Flipped Classroom Versus Traditional Textbook Instruction: Assessing Accuracy and Mental Effort at Different Levels of Mathematical Complexity. Technology, Knowledge and Learning.

[CR37] Miles MB, Huberman AM (1994). Qualitative data analysis.

[CR38] Moher D, Liberati A, Tetzlaff J, Altman DG, The Prisma Group (2009). Preferred reporting items for systematic reviews and meta-analyses: The PRISMA statement. PLOS Medicine.

[CR66] Muir Tracey, Geiger Vince (2016). The affordances of using a flipped classroom approach in the teaching of mathematics: a case study of a grade 10 mathematics class. Mathematics Education Research Journal.

[CR67] Murphy Julia, Chang Jen-Mei, Suaray Kagba (2016). Student performance and attitudes in a collaborative and flipped linear algebra course. International Journal of Mathematical Education in Science and Technology.

[CR68] Naccarato Emilie, Karakok Gulden (2015). Expectations and implementations of the flipped classroom model in undergraduate mathematics courses. International Journal of Mathematical Education in Science and Technology.

[CR39] NCSM & NCTM. (2020). *Moving forward: Mathematics learning in the era of COVID-19.* NCTM. https://www.nctm.org/uploadedFiles/Research_and_Advocacy/NCTM_NCSM_Moving_Forward.pdf

[CR69] NIELSEN PERPETUA LYNNE, BEAN NATHAN WILLIAM, LARSEN ROSS ALLEN ANDREW (2018). THE IMPACT OF A FLIPPED CLASSROOM MODEL OF LEARNING ON A LARGE UNDERGRADUATE STATISTICS CLASS. STATISTICS EDUCATION RESEARCH JOURNAL.

[CR70] Patterson Brian (2018). Flipped active learning in your mathematics classroom without videos. Primus.

[CR40] Sawchuk, S., & Sparks, S. D. (2020). Kids are behind in math because of COVID-19. Here’s what research says could help. *Education Week*. https://www.edweek.org/teaching-learning/kids-are-behind-in-math-because-of-covid-19-heres-what-research-says-could-help/2020/12

[CR71] Scott Catherine Elizabeth, Green Linda E, Etheridge Debra Lynn (2016). A comparison between flipped and lecture-based instruction in the calculus classroom. Journal of Applied Research in Higher Education.

[CR72] Şen Emine Özgür (2020). Prospective middle school mathematics teachers’ points of view on the flipped classroom: The case of Turkey.. Education and Information Technologies.

[CR73] Song Yanjie (2020). How to flip the classroom in school students’ mathematics learning: bridging in- and out-of-class activities via innovative strategies. Technology, Pedagogy and Education.

[CR41] Staker H, Horn MB (2012). Classifying K-12 blended learning.

[CR74] Steen-Utheim Anna Therese, Foldnes Njål (2018). A qualitative investigation of student engagement in a flipped classroom. Teaching in Higher Education.

[CR42] Stöhr C, Demazière C, Adawi T (2020). The polarizing effect of the online flipped classroom. Computers & Education.

[CR75] Strayer Jeremy F. (2012). How learning in an inverted classroom influences cooperation, innovation and task orientation. Learning Environments Research.

[CR76] Swart, W., Macleod, K., Mai, S., & Haytko, D. L. (2022). Resiliency During COVID-19 Disruption: Flipped vs. Traditional Classrooms. *Journal of Instructional Pedagogies, 27*, 1–20.

[CR43] Talbert R (2015). Inverting the transition-to-proof classroom. Primus.

[CR44] Tan C, Yue WG, Fu Y (2017). Effectiveness of flipped classrooms in nursing education: Systematic review and meta-analysis. Chinese Nursing Research.

[CR77] Tse Wai S., Choi Lai Y. A., Tang Wing S. (2019). Effects of video-based flipped class instruction on subject reading motivation. British Journal of Educational Technology.

[CR78] Turra Héctor (2019). Flipped classroom experiences and their impact on engineering students’ attitudes towards university-level mathematics. Higher Education Pedagogies.

[CR45] UNESCO. (2021). *Covid-19 response*. https://en.unesco.org/covid19

[CR79] Webel Corey (2018). Flipping instruction in a fifth grade class: A case of an elementary mathematics specialist.. Teaching and Teacher Education.

[CR80] Weng Pangyen (2015). Developmental Math, Flipped and Self-Paced. PRIMUS.

[CR46] Yang QF, Lin CJ, Hwang GJ (2021). Research focuses and findings of flipping mathematics classes: A review of journal publications based on the technology-enhanced learning model. Interactive Learning Environments.

[CR81] Yorganci Serpil (2020). Implementing flipped learning approach based on ‘first principles of instruction’ in mathematics courses. Journal of Computer Assisted Learning.

[CR202] Zengin Y (2017). Investigating the use of the Khan Academy and mathematics software with a flipped classroom approach inmathematics teaching. Journal of Educational Technology & Society.

