# Supplementary material for: Can flipped classroom pedagogy offer promising perspectives for mathematics education on pandemic-related issues? A systematic literature review
Source: ZDM. 2022 Jun 28;55(1):177–91. doi: 10.1007/s11858-022-01388-w (PMC9243790; doi:10.1007/s11858-022-01388-w)
Supplement: Supplementary file 1 — Supplementary file1 (PDF 6157 KB) [file 11858_2022_1388_MOESM1_ESM.pdf]

Appendix/General Overview of the Reviewed Studies, Coding Scheme (Table 5) and List of the Reviewed Studies

Can Flipped Classroom Pedagogy Offer Promising Perspectives for Mathematics Education on Pandemic-Related Issues? A Systematic Literature Review

Mustafa Cevikbas, Gabriele Kaiser
University of Hamburg

General Characteristics of the Included Studies

For this systematic review, study characteristics and methodologies of the included studies (n = 97) are presented and discussed in six categories in order to answer the first research question.

Publication Years, Academic Journals and Geographical Distribution

We did not restrict the publication year of the studies included. The analysis revealed that these studies were conducted in the last decade (see Figure 2). There was a remarkable increase in the number of articles, which have appeared in the last years, 97% of articles (n = 94) were published in 2015 or later. This publication trend shows a strong growth of interest in FC research area in recent years. The growing availability of Internet technologies (Akcayir & Akcayir, 2018), special issues on flipped mathematics classrooms (e.g., two special issues of PRIMUS in 2015), and the extraordinary circumstances that came with the COVID-19 pandemic in early 2020 may have influenced the increase in the number of publications on FC as classical learning and teaching modes have been substantially replaced by online, hybrid, or flipped pedagogy during the pandemic.

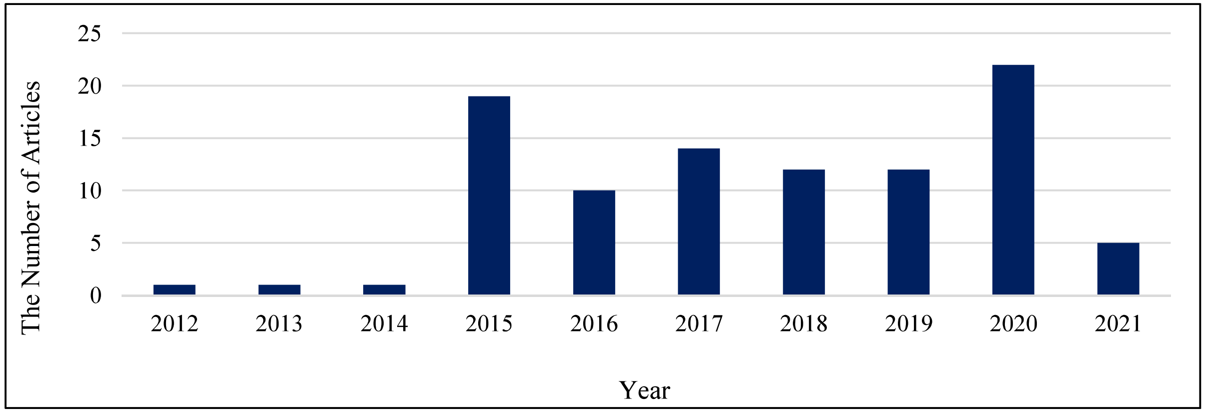

Fig. 2. The number of reviewed articles published from 2012 to July 16, 2021

Appendix/General Overview of the Reviewed Studies, Coding Scheme (Table 5) and List of the Reviewed Studies

The articles (n = 97) included in this systematic review study were published in 48 different academic journals, including journals from educational sciences (n = 18), educational technology (n = 16), mathematics education (n = 8), multidisciplinary research area (n = 4), and others (n = 2) (see Table 5). The low number of journals from mathematics education is not surprising as we only considered journals included in high-ranking databases.

As the distribution of geographical origin reflect the research trend of countries to a certain extent (Cevikbas, et al., 2021), we analyzed separately all authors' affiliations. According to our analysis, the research on the opportunities and pitfalls of FC was undertaken in 25 different countries (see Table 5) and the research contribution was dominated by researchers from the United States. The results indicated the high interest of North American (n = 97) and Asian (n = 76) researchers in FC research, comprising 77% of all studies included. Researchers from other parts of the world were underrepresented, namely Europe (14%), South America (3%), Oceania (4%), and Africa (0,4%). According to Lundin et al. (2018), dominance of North American studies might be influenced by language barriers of non-English speaking scholars. Furthermore, this result may also be related to the roots of FC pedagogy that first emerged in the North American educational research context (Bergmann & Sams, 2012).

4.1.2 Participants/Sample, Research Designs, Data Collection Methods, Duration of the Study Interventions and Frameworks of the Studies

The majority of the reviewed studies were dedicated to flipping mathematics teaching in higher education and recruited undergraduate students including pre-service teachers at university level (n = 62), followed by secondary school students (n = 19), teachers/instructors (n = 4), mixed sample of students and pre-service and in-service teachers (n = 10), and elementary school students (n = 2). There was no study focusing on the parents and school administrators as participants that is in line with the results of other influential studies (e.g., Bond, 2020). The dominance of studies from higher education might be related to the fact that researchers from higher education institutions may find it more easy to carry out a study on flipping teaching in their own institution than in other universities or in secondary schools or elementary schools as Yang et al. (2019) pointed out. Another reason may be related to the impact of PRIMUS special issues focusing only on FC studies in higher education context. Concerning the sample sizes, most of the studies (61%) included less than 100 participants although the majority of the studies (87%) were quantitively oriented studies or mixed method studies. In addition, studies were carried out with 101–200 participants (16%), followed by 201–500 participants (13%), 501–1000 participants (7%), and one study had more than 1000 participants. These results revealed that research on FC focusing elementary school students, secondary school students, and parents is needed. Additionally, more large-scale studies or studies with more participants may be favorable as a small number of participants might restrain achieving a greater statistical reliability and sustained information.

The research design of the majority of the studies followed quantitatively oriented approaches (44%) or used both quantitative and qualitative research methods (41%). However, a limited number of studies (13%) used qualitative research methods, and one study used design-based research method. According to our analysis, the studies examined used various data collection methods including surveys (78%), interviews (31%), observations (21%), questionnaires (17%), test instruments (12%), exams (11%), audio-video and screen recordings (8%), written reports/documentations such as teacher and students reflections, Learning Management System (LMS) activity reports (4%), field notes (4%), worksheets and spreadsheets (2%), and assignment (2%). More than half of the studies (58%) used multi methods for data collection (2 to 5 different methods), remaining

Appendix/General Overview of the Reviewed Studies, Coding Scheme (Table 5) and List of the Reviewed Studies

studies used only one data collection method in their research. The results indicated that there was a limited number of qualitative studies, which had the potential to provide more detailed results on the opportunities and pitfalls of FCs. Concerning the duration of study interventions, the analysis revealed that almost two-thirds (n = 59) of the studies' interventions lasted less than an academic semester (1-16 weeks), followed by 2-8 academic semesters (n= 27). 11 studies did not clarify the duration of their study interventions. In order to avoid distort the results by a strong impact of novel measures, it seems to be necessary to carry out longer-term studies.

Our analysis indicated that a limited number of studies (n=34) have clarified theoretical frameworks motivating empirical research on the potential opportunities and pitfalls of FCs, compared to 63 studies did not clearly highlight a theoretical framework used. Various theoretical frameworks (n = 26) used by researchers, including constructivist, social, cognitive, taxonomic, and motivational learning theories reflecting FCs underpinnings (see Table 5 in the Electronic Appendix). Using a theoretical framework as a lens of research can provide prolific results regarding opportunities and pitfalls of FC in mathematics education, although this makes it difficult to compare reported results within the same or similar frameworks.

Reference

Akcayir, G., & Akcayir, M. (2018). The flipped classroom: A review of its advantages and challenges. *Computers & Education, 126,* 334-345.

Bergmann, J. & Sams, A. (2012). *Flip your classroom: Reach every student in every class every day*. ISTE.

Bond, M. (2020). Facilitating student engagement through the flipped classroom approach in K-12: A systematic review. *Computers & Education, 151,* 103819.

Cevikbas, M., & Kaiser, G. (2021b). A systematic review on task design in dynamic and interactive mathematics learning environments (DIMLEs). *Mathematics, 9*(4), 399. https://doi.org/10.3390/math9040399

Cevikbas, M., Kaiser, G., & Schukajlow, S. (2021). A systematic literature review of the current discussion on mathematical modelling competencies: State-of-the-art developments in conceptualizing, measuring, and fostering. *Educational Studies in Mathematics.* https://doi.org/10.1007/s10649-021-10104-6

Lundin, M., Rensfeldt, A. B., Hillman, T., Lantz-Andersson, A., & Peterson, L. (2018). Higher education dominance and siloed knowledge: a systematic review of flipped classroom research. *International Journal of Educational Technology in Higher Education, 15*(20), 1-30.

Yang, Q. F., Lin, C. J., & Hwang, G. J. (2021). Research focuses and findings of flipping mathematics classes: a review of journal publications based on the technology-enhanced learning model. *Interactive Learning Environments, 29(*6), 905-938.

Table 5:

Coding Scheme for the Study Characteristics and Methodologies

No	Author(s)/Year	Origin Countries of Authors	Sample/Sample Size	Research Method/ Design	Framework	LMS	Journal
1	Voigt et al. (2020)	USA (3), Norway (1)	27 undergraduates	qualitative study / teaching experiment	Realistic Mathematics Education / Culturally Responsive Pedagogy	—	ZDM Mathematics Education
2	Lo & Hew (2020)	China (2)	253 secondary students and 3 teachers	design-based research	Self-Determination Theory	Moodle	Journal of Computer Assisted Learning

Appendix/General Overview of the Reviewed Studies, Coding Scheme (Table 5) and List of the Reviewed Studies

3	Sun & Xie (2020)	Denmark (1), USA (1), China (1)	104 undergraduates	quantitative	Achievement Goal Theory	Desire2Learn	The Internet and Higher Education
4	Yorganci (2020)	Turkey (1)	163 undergraduates	quantitative / quasi-experimental research	First Principles of Instruction Design Theory	Moodle and Khan Academy	Journal of Computer Assisted Learning
5	Song (2020)	China (1)	37 secondary school students	mixed method	Social Constructivism, 5-D Pedagogical Framework of the Flipped Classroom Comprising 'Discover, Diagnose, Devise, Develop and Defend'	Schoology	Technology, Pedagogy and Education
6	Muir (2020)	Australia (1)	27 secondary students and a teacher	mixed method	Self Determination Theory	—	Mathematics Education Research Journal
7	Şen & Hava (2020)	Turkey (2)	41 pre-service teachers	qualitative / basic qualitative research method	—	Edmodo	Education and Information Technologies
8	Wei et al. (2020)	China (5), Taiwan (2), USA (1)	88 secondary school students	mixed method	—	QQ	Educational Technology Research and Development
9	Belmonte et al. (2019)	Spain (4)	60 secondary school students	quantitative / experimental design of a descriptive and correlational type	—	—	Mathematics
10	Jordan et al. (2019)	Spain (3)	157 undergraduates	quantitative	—	Sakai	Education Sciences
11	Collins (2019)	USA (1)	177 undergraduates	quantitative	—	Desire2Learn	International Journal of Mathematical Education in Science and Technology
12	Fredriksen. & Hadjerrouit (2020)	Norway (2)	45 undergraduates	qualitative / a multiple case study	Activity Theory	—	International Journal of Mathematical Education in Science and Technology
13	Amstelveen (2019)	USA (1)	77 undergraduates	quantitative	—	—	Education and Information Technologies
14	Turra et al. (2019)	Chile (5)	76 undergraduates	quantitative	—	—	Higher Education Pedagogies

Appendix/General Overview of the Reviewed Studies, Coding Scheme (Table 5) and List of the Reviewed Studies

15	Chien & Hsieh (2018)	Taiwan (2)	60 undergraduates	mixed method / quasi-experimental	FLIPPED Model (Chen, et al., 2014)	Edpuzzle	International Journal of Online Pedagogy and Course Design
16	Mohamed & Lamia (2018)	Algeria (2)	50 undergraduates	quantitative	—	—	Computers & Education
17	Webel et al. (2018)	USA (3)	one teacher/instructor	qualitative / case study	—	EdPuzzle	Teaching and Teacher Education
18	Lo et al. (2018)	China (3)	382 secondary school students	mixed method	First Principles of Instruction (Merrill, 2002)	—	Computers & Education
19	Lopes & Soares (2018)	Portugal (2)	803 undergraduates	quantitative	—	Moodle	The International Journal of Management Education
20	Khan & Watson (2018)	Australia (2)	483 undergraduates	mixed method	—	—	Journal of University Teaching & Learning Practice
21	Steen-Ulheim & Foldnes (2018)	Norway (2)	12 undergraduates	qualitative	Social Constructivism	—	Teaching in Higher Education
22	Carter et al. (2018)	USA (3)	632 undergraduates	quantitative / quasi-experimental design	—	Blackboard	AERA Open
23	Li et al. (2017)	China (3)	120 undergraduates	quantitative / quasi-experimental research	—	—	EURASIA Journal of Mathematics Science and Technology Education
24	De Araujo et al. (2017a)	USA (2), Turkey (1)	21 undergraduates and a teacher	qualitative / case study	Enacted Mathematics Curriculum Framework (Remillard & Heck, 2014)	—	ZDM Mathematics Education
25	Zengin (2017)	Turkey (1)	28 undergraduates	mixed method	—	Khan Academy	Educational Technology & Society
26	Lee et al. (2017)	South Korea (3)	21 undergraduates and a teacher	mixed method	—	—	Educational Technology Research and Development
27	De Araujo et al. (2017b)	USA (2), Turkey (1)	2 teachers/instructors	mixed method	—	—	Teaching and Teacher Education
28	Foldnes (2017)	Norway (1)	241 undergraduates	quantitative	—	—	Nordic Journal of Digital Literacy
29	Johnston (2017)	Australia (1)	38 undergraduates	quantitative / sequential case study	—	—	International Journal of Mathematical Education in Science and Technology

Appendix/General Overview of the Reviewed Studies, Coding Scheme (Table 5) and List of the Reviewed Studies

30	Hwang & Lai (2017)	Taiwan (2)	45 elementary school students	quantitative / quasi-experimental study	—	—	Educational Technology & Society
31	Lo & Hew (2017b)	China (2)	154 secondary school students and a teacher	mixed method	First Principles of Instruction Design Theory	—	Educational Technology & Society
32	Song & Kapur (2017)	China (1), Switzerland (1)	50 secondary school students	mixed method	—	—	Educational Technology & Society
33	Maciejewski (2016)	New Zealand (1)	690 undergraduates	mixed method	—	—	Teaching Mathematics and Its Applications
34	Chen et al. (2016)	Taiwan (3)	265 secondary school students	mixed method	—	—	British Journal of Educational Technology
35	Lai & Hwang (2016)	Taiwan (2)	44 elementary school students	quantitative / quasi-experimental study	Self Regulated Learning	—	Computers & Education
36	Bhagat et al. (2016)	Taiwan (3)	82 secondary school students	quantitative / quasi-experimental design	The Cognitive Theory of Multimedia Learning (CTML)	—	Educational Technology & Society
37	Scott et al. (2016)	USA (3)	96 undergraduates and 2 instructors	mixed method	—	Sakai<br><br>Online homework system WebAssign	Journal of Applied Research in Higher Education
38	Naccarato & Karakok (2015)	USA (2)	19 teachers/instructors	qualitative	—	—	International Journal of Mathematical Education in Science and Technology
39	Tawfik & Lilly (2015)	USA (2)	24 undergraduates	qualitative / case study	Problem Based Learning	—	Technology, Knowledge and Learning
40	Mattis (2015)	USA (1)	48 undergraduates	quantitative / quasi-experimental design	Sweller's (1988) Cognitive Load Theory,<br><br>Mayer's (2009) Cognitive Theory of Multimedia Learning	—	Technology, Knowledge and Learning
41	Sutama et al. (2020)	Indonesia (4)	secondary school students (sample size not specified)	mixed method	—	—	Universal Journal of Educational Research
42	Ramadhani & Fitri (2020)	Indonesia (2)	105 secondary school students	quantitative	—	—	Universal Journal of Educational Research

Appendix/General Overview of the Reviewed Studies, Coding Scheme (Table 5) and List of the Reviewed Studies

43	Krouss & Lesseig (2020)	USA (2)	329 undergraduates	mixed method	—	—	PRIMUS
44	Lo & Hew (2020)	China (2)	76 secondary school students	mixed method	Merrill’s (2002) First Principles of Instruction Design Theory / Self-Determination Theory of Motivation	Moodle	Interactive Learning Environments
45	Anbalagan & Jayachithra (2020)	India (2)	60 pre-service teachers	quantitative	—	—	International Journal of Scientific & Technology Research
46	Cevikbas & Kaiser (2020)	Germany (2)	68 secondary students and a teacher	qualitative / case study	Social Constructivism	Edmodo	ZDM Mathematics Education
47	Naidoo (2020)	south africa (1)	32 teachers/instructo rs	qualitative	Activity Theory	—	Universal Journal of Educational Research
48	Fedista et al. (2019)	Indonesia (3)	secondary school students (sample size not specified)	mixed method	—	—	International Journal of Scientific & Technology Research
49	Ramadhani et al. (2019)	Indonesia (3), Japan (1)	62 secondary school students	quantitative / quasi-experimental research	Problem Based Learning	Google Classroom	Journal for the Education of Gifted Young Scientists
50	Jarrah & Diab (2019)	United Arab Emirates<br><br>UAE (2)	79 secondary school students	quantitative / quasi experimental design	—	Khan Academy	The Journal of Social Sciences Research
51	Patterson et al. (2018)	USA (3)	65 undergraduates	quantitative	—	Moodle	PRIMUS
52	Adams & Dove (2018)	USA (2)	38 undergraduates	quantitative	—	MyMathLab	PRIMUS
53	Nielsen et al. (2018)	USA (3)	365 undergraduates	quantitative / quasi-experimental design	—	—	Statistics Education Research Journal
54	Albalawi (2018)	Saudi Arabia (1)	92 undergraduates	quantitative / quasi experimental design	—	Moodle	International Journal of Research in Education and Science
55	Novak et al. (2017)	New Zealand (3)	300 undergraduates	mixed method	—	—	International Journal of Mathematical Education in Science and Technology

Appendix/General Overview of the Reviewed Studies, Coding Scheme (Table 5) and List of the Reviewed Studies

56	Talbert (2015)	USA (1)	978 undergraduates	mixed method	Self-Regulated Learning Theory	—	PRIMUS
57	Weng (2015)	USA (1)	125 undergraduates	quantitative	—	MyMathLab, Educreations	PRIMUS
58	Guerrero et al. (2015)	USA (5)	68 undergraduates	mixed method	—	—	PRIMUS
59	Anderson & Brennan (2015)	USA (2)	1000 undergraduates	quantitative	—	—	PRIMUS
60	Love et al. (2014)	USA (4)	55 undergraduates	mixed method	—	—	International Journal of Mathematical Education in Science and Technology
61	Strayer (2012)	USA (1)	50 undergraduates	mixed method	CUCEI Is Grounded in Moos' (1974) Theory	—	Learning Environments Research
62	Lesseig & Krouss (2016)	USA (2)	47 undergraduates	mixed method	—	Angel	International Journal of Mathematical Education in Science and Technology
63	Love et al. (2015)	USA (4)	60 undergraduates	quantitative / quasi-experimental design	Inquiry-Based Learning	—	PRIMUS
64	Grypp & Luebeck (2015)	USA (2)	21 secondary school students	qualitative / action research	Mertler's Nine-Step Framework (2013)	—	The Mathematics Teacher
65	Ogden (2015)	USA (1)	118 undergraduates and a teacher	qualitative	—	—	PRIMUS
66	Bagley (2020)	USA (1)	478 undergraduates	mixed method	—	—	PRIMUS
67	Hung et al. (2019	China (1), Taiwan (3)	238 secondary school students and 5 teachers	quantitative	Game-Based Learning	—	Interactive Learning Environments
68	Karjanto & Simon (2019)	South Korea (2)	310 undergraduates	mixed method	Bloom's Taxonomy, English Medium Instruction (EMI) and Technology Adaptation and Inverted Bloom's Taxonomy, EMI for Nonnative English Speakers in the CHC	icampus	Studies in Educational Evaluation

Appendix/General Overview of the Reviewed Studies, Coding Scheme (Table 5) and List of the Reviewed Studies

					Context and the Adaptation of Technology for Mathematical Instruction		
69	Price & Walker (2019)	UK (2)	2000 undergraduates	quantitative / quasi-experimental design	—	—	Studies in Higher Education
70	Tse et al. (2019)	China (3)	100 secondary school students	quantitative	—	—	British Journal of Educational Technology
71	Cho et al. (2015)	USA (3), South Kores (1)	74 undergraduates	mixed method	Cambourne's Conditions of Learning	—	Korean Journal of Mathematics
72	Wilson (2013)	USA (1)	53 undergraduates	quantitative	Fink's (2003) Taxonomy	Blackboard, Khan Academy	Teaching of Psychology
73	Lo (2017)	China (1)	130 secondary school students and 4 teachers	mixed method	—	Moodle	The Mathematics Teacher
74	Al-Abdullatif (2020)	Saudi Arabia (1)	64 secondary school students	quantitative	Self-Regulated Learning	Easyclass	Cogent Education
75	Al-Zoubi & Suleiman (2021)	Saudi Arabia (1), Jordan (1)	54 undergraduates	mixed method / explanatory sequential mixed methods	Three Worlds of Mathematics (Tall, 2008)	Blackboard	International Journal of Instruction
76	Bego et al. (2020)	USA (3)	859 undergraduates	quantitative / quasi-experimental design	—	Dyknow	International Journal of Mathematical Education in Science and Technology
77	Cilli-Turner (2020)	USA (1)	134 undergraduates	quantitative	—	—	PRIMUS
78	Dori et al. (2020)	Israel (3), USA (1)	374 undergraduates	quantitative	Project-Based Learning	—	EURASIA Journal of Mathematics Science and Technology Education
79	Fredriksen (2021)	Norway (1)	15 undergraduates	qualitative	Realistic Mathematics Education	—	International Journal of Science and Mathematics Education
80	Gouia & Gunn (2016)	United Arab Emirates (2)	81 undergraduates	quantitative and qualitative methods	—	AUS Learning Management System	Research and Practice in Technology Enhanced Learning
81	Heuett & William (2017)	USA (1)	82 undergraduates	quantitative and qualitative	—	MyMathLab (Pearson)	PRIMUS

Appendix/General Overview of the Reviewed Studies, Coding Scheme (Table 5) and List of the Reviewed Studies

82	Jeong et al. (2021)	Spain (2)	143 undergraduates	quantitative / randomized experimental design	—	—	Mathematics
83	Kennedy et al. (2015)	USA (4)	173 undergraduates	quantitative / experimental design	—	Blackboard Learn	PRIMUS
84	Kirvan et al. (2015)	USA(3)	54 secondary school students	quantitative / quasi-experimental design	—	Khan Academy, Edodo	Computers in the Schools
85	Lo & Hew (2017a)	China (2)	13 secondary school students	quantitative and qualitative methods	Merrill's (2002) First Principles of Instruction Design and Kolb's Experiential Learning Theory	—	Educational Technology & Society
86	Muir & Geiger (2016)	Australia (2)	27 secondary school students	quantitative and qualitative methods	Self-Regulated Learning and the Four Pillars of FLIP	—	Mathematics Education Research Journal
87	Murphy et al. (2016)	USA (3)	77 undergraduates	quantitative and qualitative methods	—	—	International Journal of Mathematical Education in Science and Technology
88	Overmyer (2015)	USA (1)	301 undergraduates	quantitative / quasi-experimental design	—	Blackboard	PRIMUS
89	Petrillo (2016)	USA (1)	535 undergraduates	quantitative / case study	—	—	International Journal of Mathematical Education in Science and Technology
90	Romaker (2021)	USA (1)	51 undergraduates	mixed method	Astin's (1984) Theory of Involvement	—	Community College Journal of Research and Practice
91	Cavlazoglu & Zeytuncu (2015)	USA (3)	96 undergraduates	quantitative	—	—	Journal of Educational Technology & Society
92	Salas-Rueda (2020)	Mexico (1)	61 undergraduates	mixed method	—	—	LUMAT: International Journal on Math, Science and Technology Education
93	Salas-Rueda (2021)	Mexico (1)	29 undergraduates	mixed method	—	—	Education and Information Technologies
94	Schroeder et al. (2015)	USA (3)	112 undergraduates	mixed method	—	—	PRIMUS
95	Spotts & de Blume (2020)	USA (2)	44 undergraduates	quantitative / quasi-experimental design	—	itslearning	SAGE Open

Appendix/General Overview of the Reviewed Studies, Coding Scheme (Table 5) and List of the Reviewed Studies

96	Zack et al. (2015)	USA (5)	113 undergraduates	quantitative and qualitative methods	—	—	PRIMUS
97	Ziegelmeier et al. (2015)	USA (2)	45 undergraduates	quantitative and qualitative methods	—	Moodle	PRIMUS

List of the Reviewed Studies

Adams, C., & Dove, A. (2018). Calculus students flipped out: The impact of flipped learning on calculus students' achievement and perceptions of learning. *PRIMUS, 28*(6), 600-615.

Al-Abdullatif, A. M. (2020). Investigating self-regulated learning and academic achievement in an eLearning environment: The case of K-12 flipped classroom. *Cogent Education, 7*(1), 1835145.

Albalawi, A. S. (2018). The effect of using flipped classroom in teaching calculus on students' achievements at University of Tabuk. *International Journal of Research in Education and Science, 4*(1), 198-207.

Al-Zoubi, A. M., & Suleiman, L. M. (2021). Flipped classroom strategy based on critical thinking skills: helping fresh female students acquiring derivative concept. *International Journal of Instruction, 14*(2), 791-810.

Amstelveen, R. (2019). Flipping a college mathematics classroom: An action research project. *Education and Information Technologies, 24*(2), 1337-1350.

Anbalagan, S., & Jayachithra, J. (2020). Satisfaction Of Flipped Classroom Teaching Strategies Among B. Ed Teacher Trainees. *International Journal of Scientific & Technology Research,* 9, 5196-5198.

Anderson, L., & Brennan, J. P. (2015). An experiment in “flipped” teaching in freshman calculus. *PRIMUS, 25*(9-10), 861-875.

Bagley, S. (2020). The flipped classroom, lethal mutations, and the didactical contract: A cautionary tale. *PRIMUS, 30*(3), 243-260.

Bego, C. R., Ralston, P. A., & Thompson, A. K. (2020). Improving performance in a large flipped barrier mathematics course: a longitudinal case study. *International Journal of Mathematical Education in Science and Technology,* 1-18. doi: 10.1080/0020739X.2020.1850899

Belmonte, J. L., Cabrera, A. F., Núñez, J. A. L., & Sánchez, S. P. (2019). Formative transcendence of flipped learning in mathematics students of secondary education. *Mathematics,* 7(12), 1226.

Bhagat, K. K., Chang, C. N., & Chang, C. Y. (2016). The impact of the flipped classroom on mathematics concept learning in high school. *Journal of Educational Technology & Society, 19*(3), 134-142.

Carter, C. L., Carter, R. L., & Foss, A. H. (2018). The flipped classroom in a terminal college mathematics course for liberal arts students. *AERA Open, 4*(1), 2332858418759266.

Cevikbas, M., & Kaiser, G. (2020). Flipped classroom as a reform-oriented approach to teaching mathematics. *ZDM–Mathematics Education*, 52(7), 1291-1305.

Chen, S. C., Yang, S. J., & Hsiao, C. C. (2016). Exploring student perceptions, learning outcome and gender differences in a flipped mathematics course. *British Journal of Educational Technology, 47*(6), 1096-1112.

Chien, C. F., & Hsieh, L. H. C. (2018). Exploring university students' achievement, motivation, and receptivity of flipped learning in an engineering mathematics course. *International Journal of Online Pedagogy and Course Design, 8(*4), 22-37.

Cho, H., Osborne, C., Sanders, T., & Park, K. (2015). Multidimensional teaching: Thoughtful ways of creating a flipped classroom. *The Korean Journal of Mathematics, 23*(1), 93-114.

Cilli-Turner, E. (2015). Measuring learning outcomes and attitudes in a flipped introductory statistics course. *PRIMUS, 25*(9-10), 833-846.

Collins, B. V. C. (2019). Flipping the precalculus classroom. *International Journal of Mathematical Education in Science and Technology, 50*(5), 728-746.

de Araujo, Z., Otten, S., & Birisci, S. (2017a). Teacher-created videos in a flipped mathematics class: digital curriculum materials or lesson enactments?. *ZDM–Mathematics Education, 49*(5), 687-699.

Appendix/General Overview of the Reviewed Studies, Coding Scheme (Table 5) and List of the Reviewed Studies

de Araujo, Z., Otten, S., & Birisci, S. (2017b). Mathematics teachers' motivations for, conceptions of, and experiences with flipped instruction. *Teaching and Teacher Education, 62,* 60-70.

Dori, J., Judy, Y., Kohen, Z., & Rizowy, B. (2020). Mathematics for computer science: A flipped classroom with an optional project. *EURASIA Journal of Mathematics, Science and Technology Education, 16*(12), 1-20.

Fedistia, R., Musdi, E., & Yerizon. Advantages and challenges of the flipped classroom application based learning in enhancing 10th grade senior high school students' reasoning ability. *International Journal of Scientific & Technology Research, 8*(8), 916-919.

Foldnes, N. (2017). The impact of class attendance on student learning in a flipped classroom. *Nordic Journal of Digital Literacy, 12*(1-2), 8-18.

Fredriksen, H. (2021). Exploring realistic mathematics education in a flipped classroom context at the tertiary level. *International Journal of Science and Mathematics Education, 19*(2), 377-396.

Fredriksen, H., & Hadjerrouit, S. (2020). An activity theory perspective on contradictions in flipped mathematics classrooms at the university level. *International Journal of Mathematical Education in Science and Technology, 51*(4), 520-541.

Gouia, R., & Gunn, C. (2016). Making mathematics meaningful for freshmen students: investigating students' preferences of pre-class videos. *Research and Practice in Technology Enhanced Learning, 11*(1), 1-8.

Grypp, L., & Luebeck, J. (2015). Rotating solids and flipping instruction. *The Mathematics Teacher, 109*(3), 186-193.

Guerrero, S., Beal, M., Lamb, C., Sonderegger, D., & Baumgartel, D. (2015). Flipping undergraduate finite mathematics: Findings and implications. *PRIMUS, 25(*9-10), 814-832.

Heuett, W. J. (2017). Flipping the math classroom for non-math majors to enrich their learning experience. *PRIMUS, 27*(10), 889-907.

Hung, C. Y., Sun, J. C. Y., & Liu, J. Y. (2019). Effects of flipped classrooms integrated with MOOCs and game-based learning on the learning motivation and outcomes of students from different backgrounds. *Interactive Learning Environments, 27*(8), 1028-1046.

Hwang, G. J., & Lai, C. L. (2017). Facilitating and bridging out-of-class and in-class learning: An interactive e-book-based flipped learning approach for math courses. *Journal of Educational Technology & Society, 20(*1), 184-197.

Jarrah, A. M., & Diab, K. M. A. B. M. (2019). The effect of flipped classroom model on students' achievement in the new 2016 scholastic assessment test mathematics skills. *Online Submission, 5*(3), 769-777.

Jeong, J. S., & González-Gómez, D. (2021). Flipped-OCN method in mathematics learning to analyze the attitudes of pre-service teachers. *Mathematics, 9*(6), 607.

Johnston, B. M. (2017). Implementing a flipped classroom approach in a university numerical methods mathematics course. *International Journal of Mathematical Education in Science and Technology, 48*(4), 485-498.

Jordán, C., Magreñán, Á. A., & Orcos, L. (2019). Considerations about flip education in the teaching of advanced mathematics. *Education Sciences, 9*(3), 227.

Karjanto, N., & Simon, L. (2019). English-medium instruction Calculus in Confucian-Heritage Culture: Flipping the class or overriding the culture?. *Studies in Educational Evaluation, 63*, 122-135.

Kennedy, E., Beaudrie, B., Ernst, D. C., & St. Laurent, R. (2015). Inverted pedagogy in second semester calculus. *PRIMUS, 25*(9-10), 892-906.

Khan, R. N., & Watson, R. (2018). The flipped classroom with tutor support: An experience in a level one statistics unit. *Journal of University Teaching & Learning Practice, 15*(3), 1-19.

Kirvan, R., Rakes, C. R., & Zamora, R. (2015). Flipping an algebra classroom: analyzing, modeling, and solving systems of linear equations. *Computers in the Schools, 32*(3-4), 201-223.

Krouss, P., & Lesseig, K. (2020). Effects of a flipped classroom model in an introductory college mathematics course. *PRIMUS, 30*(5), 617-635.

Lai, C. L., & Hwang, G. J. (2016). A self-regulated flipped classroom approach to improving students' learning performance in a mathematics course. *Computers & Education, 100,* 126-140.

Lee, J., Lim, C., & Kim, H. (2017). Development of an instructional design model for flipped learning in higher education. *Educational Technology Research and Development, 65*(2), 427-453.

Appendix/General Overview of the Reviewed Studies, Coding Scheme (Table 5) and List of the Reviewed Studies

Lesseig, K., & Krouss, P. (2017). Implementing a flipped instructional model in college algebra: Profiles of student activity. *International Journal of Mathematical Education in Science and Technology, 48*(2), 202-214.

Li, Y. B., Zheng, W. Z., & Yang, F. (2017). Cooperation learning of flip teaching style on the MBA mathematics education efficiency. *Eurasia Journal of Mathematics, Science and Technology Education, 13*(10), 6963-6972.

Lo, C. K. (2017). Examining the flipped classroom through action research. *The Mathematics Teacher, 110*(8), 624-627.

Lo, C. K., & Hew, K. F. (2017b). Using "First Principles of Instruction" to design mathematics flipped classroom for underperforming students. *International Journal of Learning and Teaching, 3*(2), 222-236.

Lo, C. K., & Hew, K. F. (2017c). Using "first principles of instruction" to design secondary school mathematics flipped classroom: The findings of two exploratory studies. *Journal of Educational Technology & Society, 20*(1), 222-236.

Lo, C. K., & Hew, K. F. (2020). A comparison of flipped learning with gamification, traditional learning, and online independent study: The effects on students' mathematics achievement and cognitive engagement. *Interactive Learning Environments, 28*(4), 464-481.

Lo, C. K., & Hew, K. F. (2021). Developing a flipped learning approach to support student engagement: A design-based research of secondary school mathematics teaching. *Journal of Computer Assisted Learning, 37*(1), 142-157.

Lo, C. K., Lie, C. W., & Hew, K. F. (2018). Applying "First Principles of Instruction" as a design theory of the flipped classroom: Findings from a collective study of four secondary school subjects. *Computers & Education,* 118, 150-165.

Lopes, A. P., & Soares, F. (2018). Perception and performance in a flipped Financial Mathematics classroom. *The International Journal of Management Education, 16*(1), 105-113.

Love, B., Hodge, A., Corritore, C., & Ernst, D. C. (2015). Inquiry-based learning and the flipped classroom model. *PRIMUS, 25*(8), 745-762.

Love, B., Hodge, A., Grandgenett, N., & Swift, A. W. (2014). Student learning and perceptions in a flipped linear algebra course. *International Journal of Mathematical Education in Science and Technology, 45*(3), 317-324.

Maciejewski, W. (2016). Flipping the calculus classroom: An evaluative study. *Teaching Mathematics and its Applications: An International Journal of the IMA, 35*(4), 187-201.

Mattis, K. V. (2015). Flipped classroom versus traditional textbook instruction: Assessing accuracy and mental effort at different levels of mathematical complexity. *Technology, Knowledge and Learning, 20*(2), 231-248.

Mohamed, H., & Lamia, M. (2018). Implementing flipped classroom that used an intelligent tutoring system into learning process. *Computers & Education,* 124, 62-76.

Muir, T. (2021). Self-determination theory and the flipped classroom: a case study of a senior secondary mathematics class. *Mathematics Education Research Journal, 33*(3), 569-587.

Muir, T., & Geiger, V. (2016). The affordances of using a flipped classroom approach in the teaching of mathematics: a case study of a grade 10 mathematics class. *Mathematics Education Research Journal, 28*(1), 149-171.

Murphy, J., Chang, J. M., & Suaray, K. (2016). Student performance and attitudes in a collaborative and flipped linear algebra course. *International Journal of Mathematical Education in Science and Technology, 47*(5), 653-673.

Naccarato, E., & Karakok, G. (2015). Expectations and implementations of the flipped classroom model in undergraduate mathematics courses. *International Journal of Mathematical Education in Science and Technology, 46*(7), 968-978.

Naidoo, J. (2020). Exploring the Flipped Learning Approach within a Mathematics Higher Education Milieu in the Era of the Fourth Industrial Revolution. *Universal Journal of Educational Research, 8*(6), 2542-2553.

Nielsen, P. L., Bean, N. W., & Larsen, R. A. A. (2018). The impact of a flipped classroom model of learning on a large undergraduate statistics class. *Statistics Education Research Journal, 17*(1).

Novak, J., Kensington-Miller, B., & Evans, T. (2017). Flip or flop? Students' perspectives of a flipped lecture in mathematics. *International Journal of Mathematical Education in Science and Technology, 48*(5), 647-658.

Appendix/General Overview of the Reviewed Studies, Coding Scheme (Table 5) and List of the Reviewed Studies

Ogden, L. (2015). Student perceptions of the flipped classroom in college algebra. *PRIMUS, 25*(9-10), 782-791.

Overmyer, J. (2015). Research on flipping college algebra: Lessons learned and practical advice for flipping multiple sections. *PRIMUS, 25*(9-10), 792-802.

Patterson, B., McBride, C. R., & Gieger, J. L. (2018). Flipped active learning in your mathematics classroom without videos. *PRIMUS, 28*(8), 742-753.

Petrillo, J. (2016). On flipping first-semester calculus: A case study. *International Journal of Mathematical Education in Science and Technology, 47*(4), 573-582.

Ramadhani, R., & Fitri, Y. (2020). A Project-based learning into flipped classroom for ePUB3 electronic mathematics learning module (eMLM)-based on course design and implementation. *Universal Journal of Educational Research, 8*(7), 3119-3135.

Ramadhani, R., Rofiqul, U. M. A. M., Abdurrahman, A., & Syazali, M. (2019). The effect of flipped-problem based learning model integrated with LMS-google classroom for senior high school students. *Journal for the Education of Gifted Young Scientists, 7*(2), 137-158.

Romaker, D. (2021). Benefits of flipped learning for developmental mathematics. *Community College Journal of Research and Practice*, 1-13. doi: 10.1080/10668926.2021.1919241

Price, C., & Walker, M. (2021). Improving the accessibility of foundation statistics for undergraduate business and management students using a flipped classroom. *Studies in Higher Education, 46*(2), 245-257.

Sahin, A., Cavlazoglu, B., & Zeytuncu, Y. E. (2015). Flipping a college calculus course: A case study. *Journal of Educational Technology & Society, 18*(3), 142-152.

Salas-Rueda, R. A. (2020). Flipped classroom: Pedagogical model necessary to improve the participation of the students during the learning process. *LUMAT: International Journal on Math, Science and Technology Education, 8*(1), 271-296.

Salas-Rueda, R. A. (2021). Use of flipped classroom in the marketing career during the educational process on financial mathematics. *Education and Information Technologies,* 26, 4261–4284.

Schroeder, L. B., McGivney-Burelle, J., & Xue, F. (2015). To flip or not to flip? An exploratory study comparing student performance in calculus I. *PRIMUS, 25*(9-10), 876-885.

Scott, C. E., Green, L. E., & Etheridge, D. L. (2016). A comparison between flipped and lecture-based instruction in the calculus classroom. *Journal of Applied Research in Higher Education, 8*(2), 252-264.

Song, Y. (2020). How to flip the classroom in school students' mathematics learning: Bridging in-and out-of-class activities via innovative strategies. *Technology, Pedagogy and Education, 29*(3), 327-345.

Song, Y., & Kapur, M. (2017). How to flip the classroom-"productive failure or traditional flipped classroom" pedagogical design?. *Educational Technology & Society, 20*(1), 292-305.

Spotts, J. D., & Gutierrez de Blume, A. P. (2020). A pilot study on the effect of the flipped classroom model on pre-calculus performance. *SAGE Open, 10*(4), 2158244020982604.

Sun, Z., & Xie, K. (2020). How do students prepare in the pre-class setting of a flipped undergraduate math course? A latent profile analysis of learning behavior and the impact of achievement goals. *The Internet and Higher Education, 46,* 100731.

Sutama, Prayitno, H. J., Ishartono, N., & Sari, D. P. (2020). Development of Mathematics Learning Process by Using Flipped Classroom Integrated by STEAM Education in Senior High School. *Universal Journal of Educational Research, 8*(8), 3690-3697

Steen-Utheim, A. T., & Foldnes, N. (2018). A qualitative investigation of student engagement in a flipped classroom. *Teaching in Higher Education, 23(*3), 307-324.

Strayer, J. F. (2012). How learning in an inverted classroom influences cooperation, innovation and task orientation. *Learning Environments Research, 15*(2), 171-193.

Şen, E. Ö., & Hava, K. (2020). Prospective middle school mathematics teachers' points of view on the flipped classroom: The case of Turkey. *Education and Information Technologies, 25*(5), 3465-3480.

Talbert, R. (2015). Inverting the transition-to-proof classroom. *PRIMUS, 25*(8), 614-626.

Tawfik, A. A., & Lilly, C. (2015). Using a flipped classroom approach to support problem-based learning. *Technology, Knowledge and Learning, 20*(3), 299-315.

Tse, W. S., Choi, L. Y., & Tang, W. S. (2019). Effects of video-based flipped class instruction on subject reading motivation. *British Journal of Educational Technology, 50*(1), 385-398.

Appendix/General Overview of the Reviewed Studies, Coding Scheme (Table 5) and List of the Reviewed Studies

Turra, H., Carrasco, V., González, C., Sandoval, V., & Yáñez, S. (2019). Flipped classroom experiences and their impact on engineering students' attitudes towards university-level mathematics. *Higher Education Pedagogies, 4*(1), 136-155.

Voigt, M., Fredriksen, H., & Rasmussen, C. (2020). Leveraging the design heuristics of realistic mathematics education and culturally responsive pedagogy to create a richer flipped classroom calculus curriculum. *ZDM–Mathematics Education, 52*(5), 1051-1062.

Webel, C., Sheffel, C., & Conner, K. A. (2018). Flipping instruction in a fifth grade class: A case of an elementary mathematics specialist. *Teaching and Teacher Education, 71*, 271-282.

Wei, X., Cheng, I., Chen, N. S., Yang, X., Liu, Y., Dong, Y., & Zhai, X. (2020). Effect of the flipped classroom on the mathematics performance of middle school students. *Educational Technology Research & Development, 68*(3), 1461–1484.

Weng, P. (2015). Developmental math, flipped and self-paced. *PRIMUS, 25*(9-10), 768-781.

Wilson, S. G. (2013). The flipped class: A method to address the challenges of an undergraduate statistics course. *Teaching of Psychology, 40(*3), 193-199.

Yorganci, S. (2020). Implementing flipped learning approach based on 'first principles of instruction' in mathematics courses. *Journal of Computer Assisted Learning, 36*(5), 763-779.

Zack, L., Fuselier, J., Graham-Squire, A., Lamb, R., & O'Hara, K. (2015). Flipping freshman mathematics. *PRIMUS, 25*(9-10), 803-813.

Zengin, Y. (2017). Investigating the use of the Khan Academy and mathematics software with a flipped classroom approach in mathematics teaching. *Journal of Educational Technology & Society, 20*(2), 89-100.

Ziegelmeier, L. B., & Topaz, C. M. (2015). Flipped calculus: A study of student performance and perceptions. *PRIMUS, 25(*9-10), 847-860.
